# Observations of a Solar Energetic Particle Event From Inside and Outside the Coma of Comet 67P

**DOI:** 10.1029/2022JA030398

**Published:** 2022-11-29

**Authors:** A. Wellbrock, G. H. Jones, N. Dresing, A. J. Coates, C. Simon Wedlund, H. Nilsson, B. Sanchez‐Cano, E. Palmerio, L. Turc, M. Myllys, P. Henri, C. Goetz, O. Witasse, T. A. Nordheim, K. Mandt

**Affiliations:** ^1^ Mullard Space Science Laboratory University College London London UK; ^2^ The Centre for Planetary Science at UCL/Birkbeck London UK; ^3^ Department of Physics and Astronomy Turku Collegium for Science, Medicine and Technology University of Turku Turku Finland; ^4^ Space Science Institute Austrian Academy of Sciences Vienna Austria; ^5^ Swedish Institute of Space Physics Kiruna Sweden; ^6^ Department of Computer Science, Electrical and Space Engineering Luleå University of Technology Kiruna Sweden; ^7^ School of Physics and Astronomy Planetary Science Group University of Leicester Leicester UK; ^8^ Predictive Science Inc. San Diego CA USA; ^9^ Department of Physics University of Helsinki Helsinki Finland; ^10^ LPC2E CNRS Université d'Orléans OSUC CNES Orléans France; ^11^ Laboratoire Lagrange Observatoire de la Côte d'Azur Université Côte d'Azur CNRS Nice France; ^12^ ESTEC European Space Agency Noordwijk The Netherlands; ^13^ Jet Propulsion Laboratory California Institute of Technology Pasadena CA USA; ^14^ Johns Hopkins Applied Physics Laboratory Laurel MD USA

**Keywords:** comets, ionospheres, planetary bow shocks, energetic particles, interactions with solar wind plasma and fields, coronal mass ejections

## Abstract

We analyze observations of a solar energetic particle (SEP) event at Rosetta's target comet 67P/Churyumov‐Gerasimenko during 6–10 March 2015. The comet was 2.15 AU from the Sun, with the Rosetta spacecraft approximately 70 km from the nucleus placing it deep inside the comet's coma and allowing us to study its response. The Eastern flank of an interplanetary coronal mass ejection (ICME) also encountered Rosetta on 6 and 7 March. Rosetta Plasma Consortium data indicate increases in ionization rates, and cometary water group pickup ions exceeding 1 keV. Increased charge exchange reactions between solar wind ions and cometary neutrals also indicate increased upstream neutral populations consistent with enhanced SEP induced surface activity. In addition, the most intense parts of the event coincide with observations interpreted as an infant cometary bow shock, indicating that the SEPs may have enhanced the formation and/or intensified the observations. These solar transient events may also have pushed the cometopause closer to the nucleus. We track and discuss characteristics of the SEP event using remote observations by SOHO, WIND, and GOES at the Sun, in situ measurements at Solar Terrestrial Relations Observatory Ahead, Mars and Rosetta, and ENLIL modeling. Based on its relatively prolonged duration, gradual and anisotropic nature, and broad angular spread in the heliosphere, we determine the main particle acceleration source to be a distant ICME which emerged from the Sun on 6 March 2015 and was detected locally in the Martian ionosphere but was never encountered by 67P directly. The ICME's shock produced SEPs for several days which traveled to the in situ observation sites via magnetic field line connections.

## Key Points

- We track a solar energetic particle (SEP) event in situ at Solar Terrestrial Relations Observatory Ahead, Mars and Rosetta and determine the main source to be a non‐local interplanetary coronal mass ejection shock distant to 67P
- Increased solar wind charge exchange at 67P may indicate an increase in upstream neutral populations and associated SEP induced sputtering
- The most intense parts of the SEP event at 67P coincide with observations of an infant bow shock and may have enhanced its formation

## 1 Introduction

Comets are considered left‐over material from the formation of the Solar System and can therefore provide a unique window into the conditions that were present then. A comet's coma and tails are formed as a result of volatiles near the surface being heated, sublimated, and outgassed due to Solar radiation thermal forcing, which increases with increasing proximity to the Sun. This cometary gas and dust can extend out to millions of km from the nucleus because of the comet's relatively small size (typically a few km) and corresponding weak gravity. Ionization of outgassed cometary neutral particles occurs by solar ultraviolet radiation, electron impact ionization, charge exchange with solar wind particles, and marginally from direct solar wind ionization. Once the neutrals are ionized, they start to feel the motional electric field of the solar wind and become pickup ions, modifying the surrounding solar wind by processes such as mass‐loading. Due to the conservation of angular momentum, this leads to a slowing of the plasma near the nucleus, and a magnetic pile‐up; magnetic field lines drape around the obstacle because the interplanetary magnetic field is frozen in. This type of interaction shares some similarities with the solar wind interaction at unmagnetized planets such as Venus. When the comet is sufficiently active, a bow shock can form to slow down the supersonic solar wind (e.g., at comet 1P/Halley, Galeev et al., 1986), forming the first boundary between the external and cometary plasma, similar to bow shocks at unmagnetized planets. Gunell et al. (2018) reported the first two sets of observations of an infant bow shock at the Rosetta mission's target comet 67P/Churyumov‐Gerasimenko, one of which coincides with the event studied here on 7 March 2015. They interpret this to be an early stage of a solar wind deflection structure predicted by models (e.g., Behar et al., 2018) that will evolve into a fully developed cometary bow shock. Goetz et al. (2021) surveyed the complete Rosetta comet phase and identified over 300 events consistent with simulations of this structure.

The Rosetta spacecraft traveled alongside 67P from August 2014 until 30 September 2016, that is, during solar cycle 24 from shortly after solar maximum until well into the declining phase. This time period included the comet's approach toward the Sun from about 3.6 AU, perihelion at 1.24 AU, and part of its journey back out toward Jupiter's orbit (Taylor et al., 2017); the mission ended at 3.8 AU. Rosetta's motion around the comet occurred at walking pace (∼1 m s^−1^) and covered many different cometary latitudes and longitudes (mostly deep inside the coma), in addition to a large range of solar illumination conditions. 67P is a mildly active comet (e.g., Coates, 2016) with a gas production rate of 10^25^–10^29^ s^−1^ (Hansen et al., 2016) which is highly dependent on factors such as heliocentric distance. Similarly, neutral and plasma densities during the Rosetta mission spanned 10^6^–10^9^ and 10^1^–10^4^ cm^−3^, respectively (e.g., Heritier et al., 2018).

Solar transient events such as coronal mass ejections (CMEs), corotating interaction regions (CIRs), solar flares, and solar energetic particle (SEP) events can have significant effects on cometary environments such as global or local increases in ionization rates, the removal of neutrals, and dust charging. Before the Rosetta era, data were only available from comet flybys and remote observations. Therefore few studies of the effects of solar transient events were possible, such as Russell et al. (1987), who examined possible solar wind sources for the sudden brightening of comet C/1983 H1 (Iras‐Araki‐Alcock). The Rosetta mission allows us to study the effects of solar transient events from deep inside the cometary coma for the first time. Any responses to such events will be modified by the dense coma. Rosetta studies include, for example, Noonan et al. (2018) who report large increases in atomic UV emission from 67P during a CME impact on 5/6 October 2015 using the Rosetta Alice UV spectrograph. Edberg, Alho, et al. (2016) describe observations during this event using Rosetta Plasma Consortium (RPC) data such as a denser plasma environment, plasma density increases by a factor of 10 reaching up to 600 cm^−3^ (and therefore also affecting neutral‐ion ratios), and magnetic field enhancements up to 40–100 nT including individual spikes reaching 200 nT. We note that during this event Rosetta was performing a dayside excursion at a distance of 800 km from the comet nucleus, placing it in the outer coma (ion pile‐up) region which is much further away from the nucleus than its nominal orbit. Similarly, Goetz et al. (2019) detail extreme (up to 300 nT) magnetic pile‐up observations associated with an impact of a CME combined with a CIR, which is an increase of six times the already draped and enhanced nominal magnetic field in the coma. Hajra et al. (2018) and Edberg, Eriksson, et al. (2016) also studied CIRs and their effects on the comet, where Hajra et al. (2018) report plasma density enhancements of 500%–1000% associated primarily with a strong increase in electron impact ionization. One of the CIRs studied by Edberg, Eriksson, et al. (2016) impacted 67P on 22 October 2014 together with a massive interplanetary coronal mass ejection (ICME). This ICME was investigated and traced through the Solar System by Witasse et al. (2017). On the other hand, Edberg et al. (2019) investigated the direct effects of several solar flares on 67P's plasma environment and report that in most cases, flares do not seem to significantly increase the plasma density. In addition, Myllys et al. (2021) discuss electric field emissions related to Langmuir waves at 67P with some focus on solar transient event dependence, including the event of interest in this study in early March 2015.

Honig et al. (2019) analyzed data from the European Space Agency's (ESA's) Standard Radiation Environment Monitors (SREM) carried on several ESA spacecraft such as Rosetta and INTEGRAL in order to characterize galactic cosmic rays (GCRs) in the inner heliosphere. They note that during Rosetta's time at 67P an 8% reduction in the GCR flux was detected. Gronoff et al. (2020) also modeled the energy deposition by GCRs and SEPs in cometary nuclei, and Wurz et al. (2015) analyzed solar wind induced sputtering at 67P.

SEP events are solar transient events which consist of electrons and ions from the Sun that have been accelerated to energies in the keV, MeV, and sometimes even GeV range (e.g., Reames (1999), Reames (2013), and Desai and Giacalone (2016)). They can travel far into interplanetary space along magnetic field lines such as those following the Parker spiral. The two most common source regions are regions of magnetic reconnection at solar flare sites and shock fronts driven by CMEs or their interplanetary counterparts (ICMEs). SEP events originating from solar flares are generally classified as “impulsive”: The acceleration area is relatively small and hence only a limited number of magnetic field lines are connected to this region, which means that the interplanetary spread of these SEPs is relatively narrow. SEP events which originate at CME shocks are referred to as “gradual” events because the particles are being accelerated and propagated outwards for a longer period of time and therefore cause more gradual intensity‐time profiles; their spread in the interplanetary medium tends to be much broader due to a larger acceleration area with many magnetic connections to a much wider range of destinations. Impulsive SEP events typically extend in a relatively narrow cone, approximately 30°–60° in solar longitude, while gradual events often extend over 100°–180° (Cohen et al., 2021; L. Wang et al., 2012). There is not always a clear distinction between the two types, and SEPs generated both at flare sites and CME shock fronts can be observed as part of the same SEP event (Cane et al., 2006). Furthermore, studies such as Dresing et al. (2012) and Dresing et al. (2014, 2018) suggest the existence of alternative processes to shocks which can cause widespread SEP events, for example, effective particle transport perpendicular to the mean magnetic field.

In this study we use Rosetta's SREM instrument to identify an SEP event at 67P when the comet was between the orbits of Mars and Jupiter at 2.15 AU from the Sun, and Rosetta at a cometocentric distance of about 70 km placing it deep inside the coma. Section 2 briefly describes the main missions and instruments used in this study. The remainder of this manuscript is then organized in two parts: Sections 3 and 4. Section 3 describes and discusses in situ observations by Rosetta instruments deep inside the comet coma during the SEP event in order to learn more about its effects. In Section 4 we then study and discuss observations upstream of the comet in order to identify the source of the SEP event. We use ENLIL simulations and other in situ spacecraft observations by Solar Terrestrial Relations Observatory Ahead (STEREO A), Mars Odyssey, Mars Express and Mars Atmosphere and Volatile Evolution (MAVEN) to understand the event's wider relevance in the inner heliosphere. We show that the source of the energetic particles is linked to two solar flares that took place on 6 March 2015 and associated CMEs whose shock fronts accelerated particles for several days. Particle acceleration by ICME shocks is typically after the end of a flare therefore this process is purely driven by the shock at this stage. These particles were therefore not produced in the comet's vicinity, but traveled to the 67P environment from a remote source most likely via magnetic field line connections.

## 2 Instrumentation

ESA's Standard Radiation Environment Monitor (SREM) (Evans et al., 2008; Siegl et al., 2009) is an ESA engineering instrument designed to monitor the radiation environment which may affect the science payload instruments. Identical designs of this instrument have been fitted on several ESA spacecraft such as Herschel, Integral, PROBA‐1 and Rosetta. It consists of three detectors: one single silicon diode detector (D3) and two silicon diodes (D1/D2) arranged in a telescope configuration. The lower energy thresholds are approximately 0.5 MeV for electrons and approximately 10 MeV for protons. Table 1 lists all SREM energy channels; the energies are integrated and include protons and electrons.

**Table 1:** European Space Agency's Standard Radiation Environment Monitor (SREM) Energy Channel Table (Georgoulis et al., 2018)

SREM bin	Proton energy (MeV)		Electron energy (MeV)	
	*E*_min_	*E*_max_	*E*_min_	*E*_max_
TC1	27	*∞*	2.00	*∞*
S12	26	*∞*	2.08	*∞*
S13	27	*∞*	2.23	*∞*
S14	24	542	3.20	*∞*
S15	23	434	8.18	*∞*
TC2	49	*∞*	2.80	*∞*
S25	48	270	–	–
C1	43	86	–	–
C2	52	278	–	–
C3	76	450	–	–
C4	164	*∞*	8.10	*∞*
TC3	12	*∞*	0.80	*∞*
S32	12	*∞*	0.75	*∞*
S33	12	*∞*	1.05	*∞*
S34	12	*∞*	2.08	*∞*

**Figure 1:**
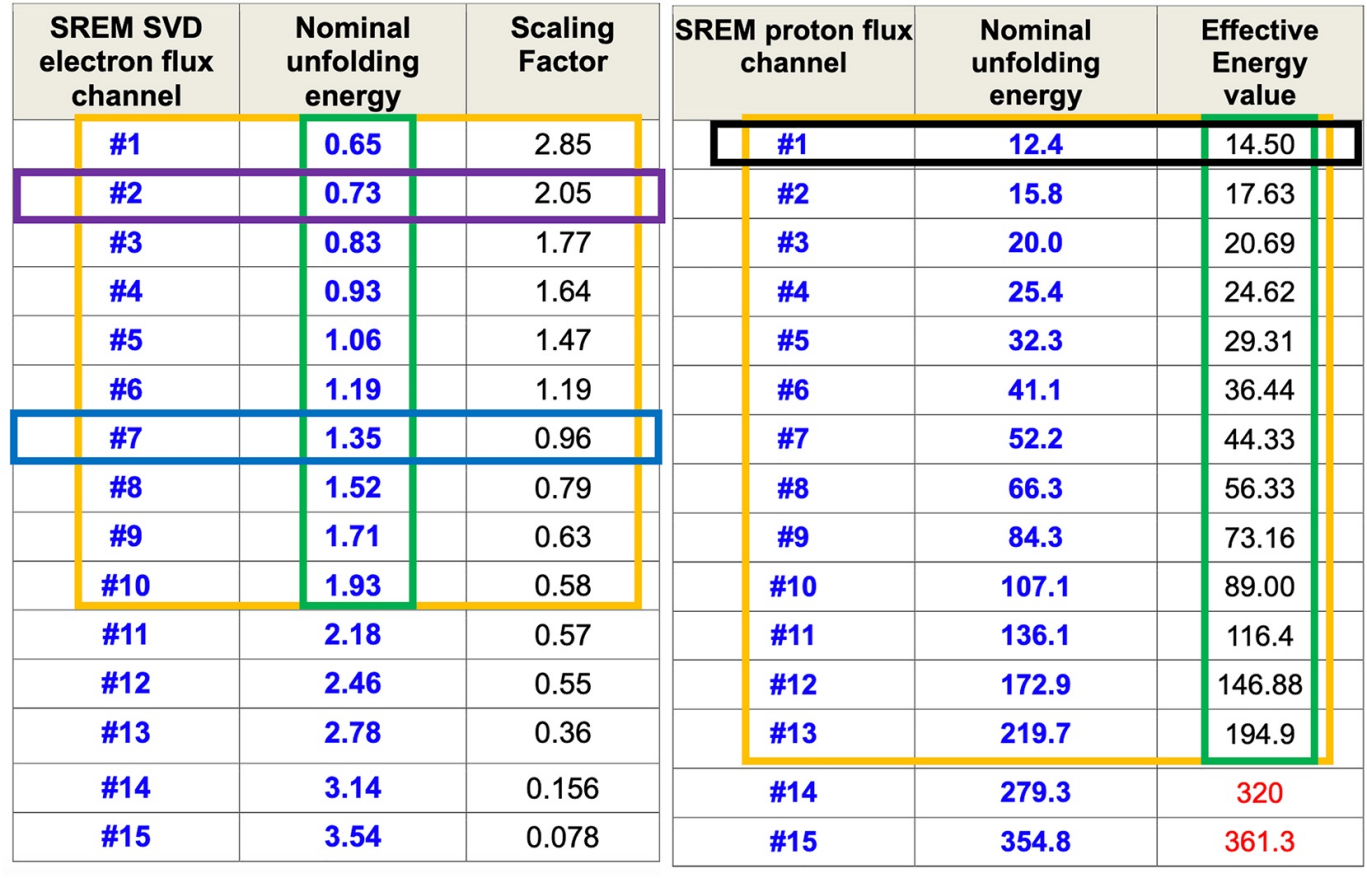
Calibrated Standard Radiation Environment Monitor (SREM) flux energy chart. The tables on the left and right outline energy information about the electron and proton channels, respectively. Due to low quality data, only the top 10 electrons channels and the top 13 proton channels can be used, as marked by the yellow boxes. The green boxes indicate the effective energy of the channel in MeV. For example, electron CH2, marked by the purple rectangle, has an effective energy of 0.73 MeV, and proton CH1, marked by the black rectangle, has an effective energy of 14.5 MeV. Figure adapted from https://pdssbn.astro.umd.edu/holdings/ro-x-srem-2-cr5-v1.0/document/sremdc_final_report_v1.0.pdf.

In this study we use raw SREM particle count rates, but also converted particle fluxes using a method by Sandberg et al. (2012) based on Singular Value Decomposition. This calibrated data set includes separate proton and electron channels which are outlined in Figure 1 (also see Institute of Accelerating Systems and Applications SREM and REM Data Consolidation final report July 2017: https://pdssbn.astro.umd.edu/holdings/ro-x-srem-2-cr5-v1.0/document/sremdc_final_report_v1.0.pdf).

The RPC instrument (Carr et al., 2007) on board the Rosetta spacecraft consists of several plasma sensors. The Ion and Electron Sensor (RPC‐IES, Burch et al., 2007) measures ion and electron flux as a function of energy (4.3 eV q^−1^–17.7 keV q^−1^) with a resolution of ΔE/E = 8% using a pair of stacked, toroidal electrostatic analyzers. In addition to studying electrons and ions within the instrumental energy range, we also use IES to study penetrating radiation. This is caused by particles with energies much higher than the instrument range (typically >100s keV) which pass through the detector and are recorded in all energy channels. For the Cassini CAPS Electron Spectrometer (Young et al., 2004), a somewhat similar electrostatic analyzer, the lower energy threshold was determined to be approximately 0.8 MeV (Rymer et al., 2001). Even though we do not know the precise energies of these particles, we can use the measurements as a proxy for SEP intensities.

Other RPC instruments we use include the top‐hat ion mass spectrometer Ion Composition Analyzer (RPC‐ICA) which measures mass‐separated energy distribution functions of positive solar wind and cometary ions from a few eV q^−1^–40 keV q^−1^ in 96 channels with an energy resolution of ΔE/E = 7% (Nilsson et al., 2007, 2017). RPC's fluxgate magnetometer (RPC‐MAG, Glassmeier et al., 2007) measures the magnetic field vector and the data used in this study were processed and calibrated according to Goetz et al. (2016). The Mutual Impedance Probe (RPC‐MIP, Trotignon et al., 2007) provides (a) the local plasma density data by probing the plasma frequency and (b) the 1D electric field in the few kHz–few MHz range. We use (a) in this study and (b) was used by Myllys et al. (2021) to study electric field emissions related to Langmuir waves including the event studied here.

We also use measurements from In situ Measurements of Particles And CME Transients (IMPACT, Luhmann et al., 2008) aboard STEREO A (Kaiser et al., 2008) to characterize the SEP event. This includes the Solar Electron and Proton Telescope (SEPT, Müller‐Mellin et al., 2008), which measures electrons from 45 to 425 keV and ions from 0.1 to 6.5 MeV in four different viewing directions and therefore allows us to determine the anisotropy of the particle event, and the Low Energy Telescope (LET, Mewaldt et al., 2008) and High Energy Telescope (HET, von Rosenvinge et al., 2008).

We use Mars Odyssey (Saunders et al., 2004), Mars Express (Chicarro et al., 2004), and the MAVEN (Jakosky, Lin, et al., 2015) SEP instrument (Larson et al., 2015) data to investigate SEP signatures of the event at Mars. Mars Odyssey's High Energy Neutron Detector (HEND, Zeitlin et al., 2010) consists of five sensors, one of which is a scintillator block made of an inner stilbene crystal surrounded by an outer cesium iodide detector. In this study we use this outer scintillator because it is the most sensitive to energetic charged particles. It is used to correct the background induced in the other sensors due to secondary particles, such as neutrons, generated in the instrument by GCRs and other energetic particles rather than from atmospheric secondary particles (Jiggens et al., 2019; Sánchez‐Cano et al., 2018). Therefore, these HEND observations are solid indirect measurements (or proxies) of SEP and GCR events. The instrument records these secondary particles in 16 different channels of increasing energy. Because it is not measuring the particles directly, we do not know the precise energy range and particle type, however the energy range is estimated to be between 30 keV and 1 MeV as calibrated by Livshits et al. (2017). In this study, we use only the most energetic channels that are primarily sensitive to particles between 195 keV and 1 MeV. In addition, Mars Express's Analyzer of Space Plasma and Energetic Atoms (ASPERA‐3, Barabash et al., 2006) Ion Mass Analyzer (IMA) is a top hat electrostatic analyzer measuring the flux of ions in the energy range 0.01–36 keV q^−1^ with ΔE/E = 7% which we use to analyze penetrating radiation, that is, particles with energies much higher than the instrument range, in a similar way to RPC‐IES as described above.

## 3 Inside the Comet Coma

### 3.1 Energetic Particle Observations

**Figure 2:**
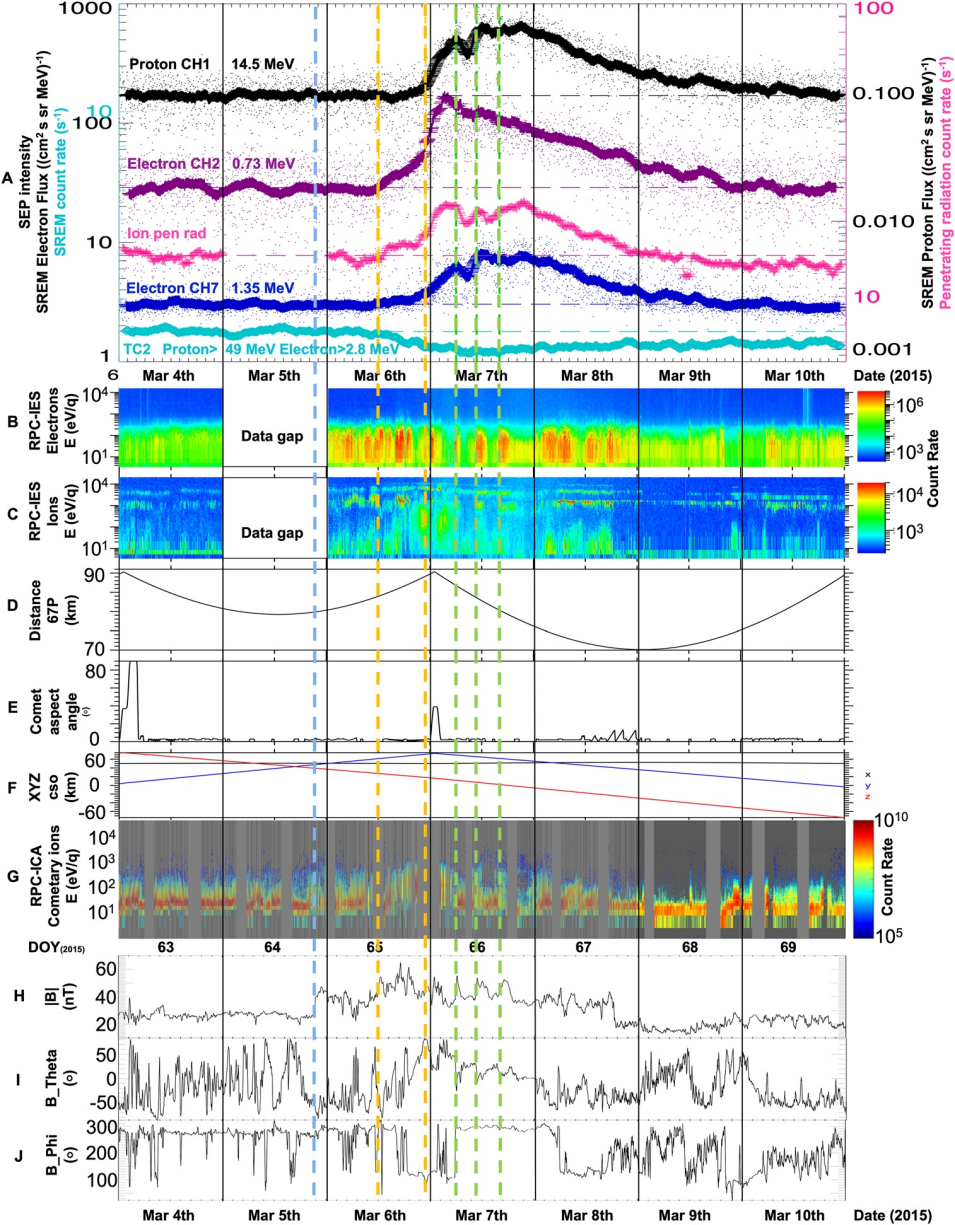
Rosetta data spanning 4–10 March 2015 (Day Of Year [DOY] 63–69). Dates shown in date (Month DD) and DOY format. A (top panel): Rosetta Standard Radiation Environment Monitor (SREM) data: channel TC2 (turquoise, raw proton >49 MeV and electron >2.8 MeV count rates), calibrated SREM fluxes in cm^2^ sr s MeV^−1^ (black: protons, 14.5 MeV, magenta: electrons, 0.73 MeV, and dark blue: electrons, 1.35 MeV, also see Table 1 and Figure 1), and Rosetta Plasma Consortium‐Ion and Electron Sensor (RPC‐IES) ion penetrating radiation count rate (pink). Horizontal dashed lines indicate mean background level of respective energy channels before solar energetic particle (SEP) event. (b) RPC‐IES electron spectrogram. (c) RPC‐IES ion spectrogram. (d) Rosetta—comet distance in km. (e) Comet aspect angle to demonstrate Rosetta's changing attitude. (f) Rosetta orbiter spacecraft position in comet solar orbital coordinates, where *x* points toward the Sun, *z* is perpendicular to *x* and projected onto the vector of the orbital plane and *y* completes the right‐handed system. *y* also points approximately in the opposite direction of the comet's orbital velocity vector. (g) Rosetta RPC‐Ion Composition Analyzer mass‐separated species spectrogram showing cometary ions only. Panels (h–j) RPC‐magnetometer 1‐min resolution data smoothed using a running 30‐point mean, in angular co‐ordinates. (h) Magnetic field strength (nT), (i) Theta (azimuth), and (j) Phi (elevation). All panels: The blue vertical dashed line indicates the arrival of interplanetary coronal mass ejection (ICME) *A*'s shock. The yellow vertical dashed lines mark approximate SEP event onset times for electron CH2 and CH7, penetrating radiation (first line, 6 March, 11:59 UT) and proton CH1 (second line, 6 March, 22:48 UT). The green vertical dashed lines mark time stamps of Gunell et al. (2018)'s infant bow shock crossings at 05:53, 10:38, and 15:54 UT on 7 March.

**Figure 3:**
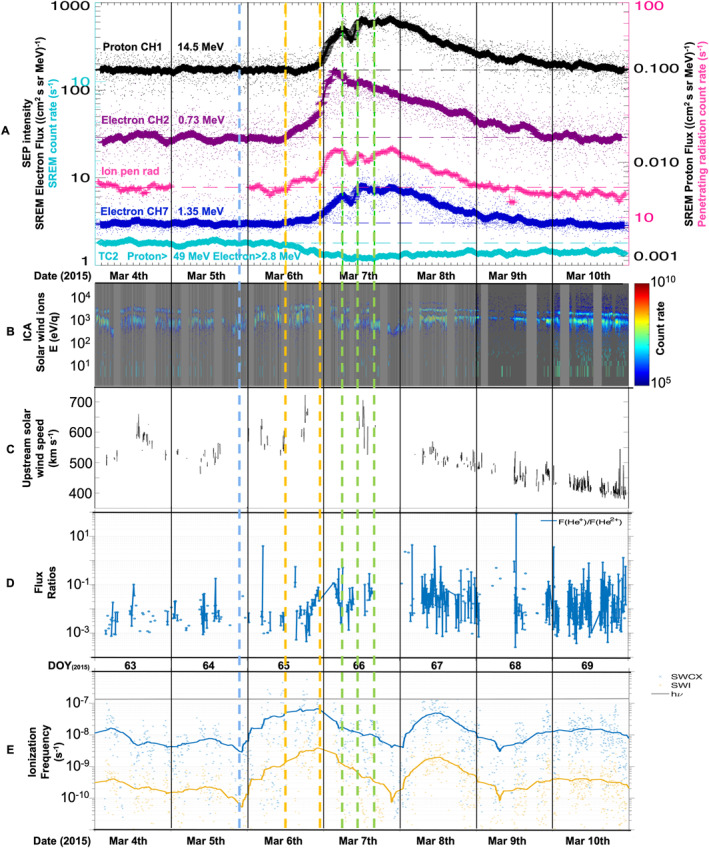
Rosetta data spanning 4–10 March 2015 (Day Of Year [DOY] 63–69). Dates are shown in date (Month DD) and DOY format. Time period is identical to Figure 2. (a) Same panel as Figure 2a. Rosetta Standard Radiation Environment Monitor (SREM) data: channel TC2 (turquoise, raw proton >49 MeV and electron >2.8 MeV count rates), calibrated SREM fluxes in cm^2^ sr s MeV^−1^ (black: protons, 14.5 MeV, magenta: electrons, 0.73 MeV, and dark blue: electrons, 1.35 MeV). Pink: Rosetta Plasma Consortium‐Ion and Electron Sensor (RPC‐IES) ion penetrating radiation. (b) Rosetta RPC‐Ion Composition Analyzer (ICA) mass‐separated species spectrogram showing solar wind ions only. (c) Upstream solar wind speed inferred from Rosetta RPC‐ICA observations of the difference between the speed of protons and alpha particles at the observation point. (d) Calculated He^+^ to He^++^ flux ratios assuming a heliocentric distance of 2 AU using RPC‐ICA data. (d) Calculated ionization frequencies using RPC‐ICA data due to: solar wind charge exchange (SWCX, blue), solar wind ionization (SWI: H^+^, He^++^, yellow), and constant photoionization (h*ν*, gray). For SWCX and SWI, a running mean for the considered time interval is shown over a 6‐hr span (solid lines). All panels: The blue vertical dashed line indicates the arrival of interplanetary coronal mass ejection (ICME) *A*'s shock. The yellow vertical dashed lines mark approximate solar energetic particle event onset times for electron CH2 and CH7, penetrating radiation (first line, 6 March, 11:59 UT) and proton CH1 (second line, 6 March, 22:48 UT). The green vertical dashed lines mark time stamps of Gunell et al. (2018)'s infant bow shock crossings at 05:53, 10:38, and 15:54 UT on 7 March.

**Figure 4:**
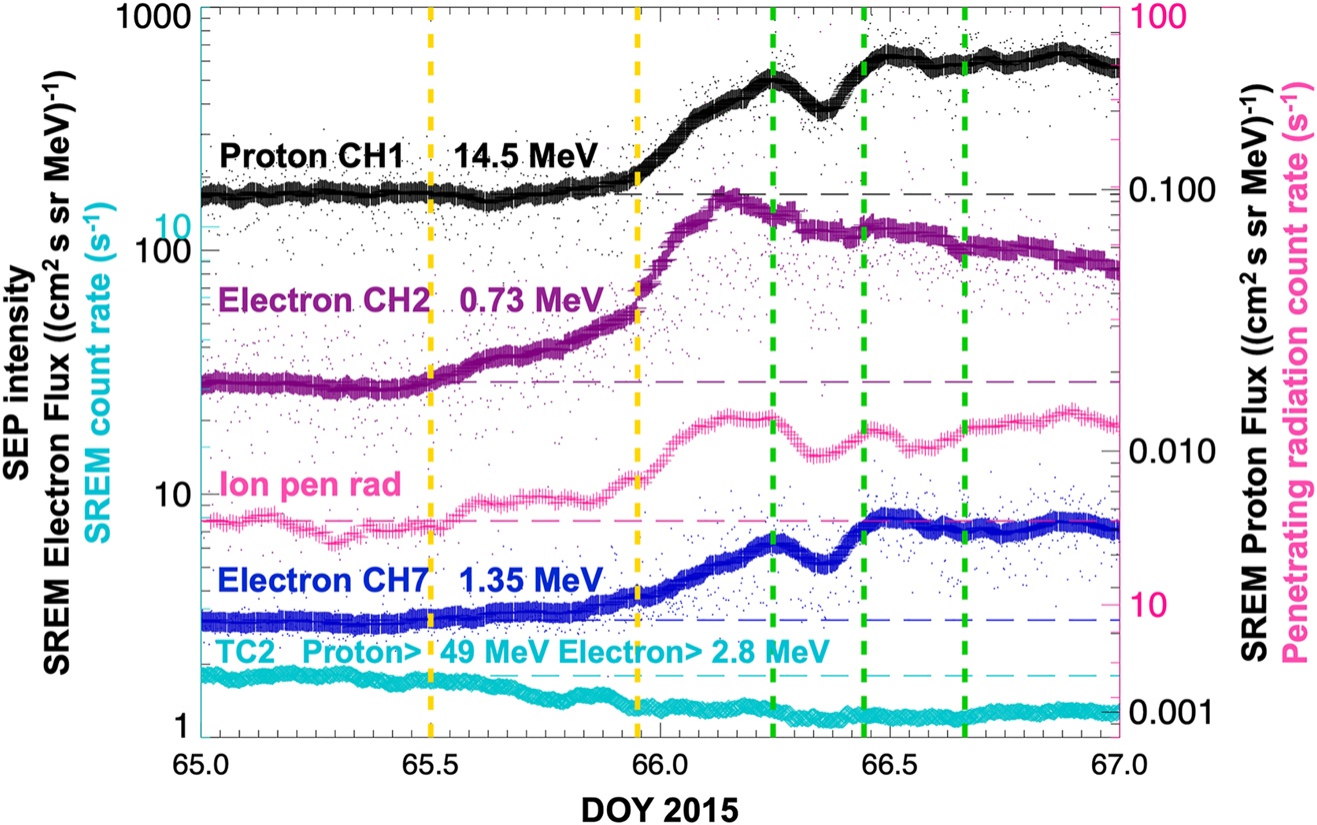
Zoomed‐in version of Figure 2a showing the onset of the solar energetic particle (SEP) event on 6 and 7 March 2015 (Day Of Year 65 and 66). Rosetta Standard Radiation Environment Monitor (SREM) data: channel TC2 (turquoise, raw proton >49 MeV and electron >2.8 MeV count rates), calibrated SREM fluxes in cm^2^ sr s MeV^−1^ (black: protons, 14.5 MeV, magenta: electrons, 0.73 MeV, and dark blue: electrons, 1.35 MeV), and Rosetta Plasma Consortium‐Ion and Electron Sensor (RPC‐IES) ion penetrating radiation (pink). The yellow vertical dashed lines mark approximate SEP event onset times for electron CH2 and CH7, penetrating radiation (first line, 6 March, 11:59 UT) and proton CH1 (second line, 6 March, 22:48 UT). The green vertical dashed lines mark time stamps of Gunell et al. (2018)'s infant bow shock crossings at 05:53, 10:38, and 15:54 UT on 7 March.

In this subsection we describe energetic particle measurements shown in Figures 2, 3, and 4, using Rosetta's radiation monitor SREM, and penetrating radiation as proxy data from Rosetta's RPC‐IES in order to characterize the SEP event at comet 67P.

The black, magenta, and dark blue traces in Figure 2a show calibrated Rosetta SREM energetic particle fluxes during 4–10 March 2015 with energies and type of particles shown as indicated. The scattered (fainter) data points are the original SREM fluxes; the solid line plots are smoothed data using a running 61‐point box car smoothing function. These three channels detected a noticeable flux increase over 3–4 days shown on this 7‐day plot.

The turquoise trace, labeled as TC2, is an additional SREM channel. Due to higher noise levels, calibrated fluxes for this channel are not available and data shown are raw count rates. Despite the data being lower quality, this channel demonstrates another important behavior. The channel covers a relatively high energy range (protons ≥49 MeV and electrons ≥2.8 MeV) and shows a slight decrease in count rates during and a few days after the event. We identify this as a Forbush decrease which is discussed further below.

Also shown in Figure 2a is penetrating radiation observed by Rosetta's RPC‐IES ion instrument, in pink. In this case a running 10‐point box car smoothing function was used. Penetrating radiation is caused by particles with energies much higher than the instrument range (typically >100s keV) passing through the detectors, and therefore consistent with energetic particles due to an SEP event which can be used as an additional marker for SEP intensity. The penetrating radiation shown in pink can also be seen in the RPC‐IES ion spectrogram in Figure 2c as bright background in all energy channels (in this case a bright light blue). Similarly, there is evidence of penetrating radiation in the IES electron spectrogram (Figure 2b), visible especially at the higher energies as light blue background enhancements. The penetrating radiation shown in pink in Figure 2a is RPC‐IES ion counts summed over the instrument's highest three energy bins. Note that the penetrating radiation can be observed in all energy bins as background counts, however we use the highest energy bins because most other channels show other features where electrons and ions in the instrument's actual energy range are present and therefore dominate over the penetrating radiation measurements.

The IES ion penetrating radiation in Figure 2a shows a clear enhancement during the event, similar in shape and duration to the SREM flux channels shown. The IES electron penetrating radiation spectrum (not shown) is very similar to the ion penetrating radiation data shown. The horizontal dashed lines in Figure 2a show the mean background level of the respective energy channels before the SEP event (DOY 63–64.5). The vertical dashed yellow lines are approximate time stamps (determined by eye) marking the SEP event onset for the electron channels and the penetrating radiation at 12:00 UT on 6 March 2015, and for the proton channel at 22:48 UT, which we refer to below. On 5 March 2015, the Eastern flank of a massive ICME driven interplanetary shock arrived at comet 67P, followed by its ICME ejecta and another ICME. According to ENLIL modeling (http://helioweather.net/), these two ICMEs were likely about to collide at STEREO A and started to interact and merge near Rosetta. We refer to these ICMEs as *A* and *B* in this study, and the combined ICME structure as ICME *AB* which is discussed further below (Sections 3.2.3 and 4, also see Table 2, and Figures 7 and 8). The vertical blue dashed line in Figures 2 and 3 marks the arrival of ICME *AB*'s shock at 21:25 UT.

Gunell et al. (2018) describe observations which they interpret as Rosetta crossing an early stage bow shock during two periods, including 7 March 2015, that is, coinciding with the most intense part of the SEP event described here. The green dashed lines correspond to Gunell et al. (2018)'s observations of infant bow shock crossings marked by cyan ticks in their Figure 1 at 05:53, 10:38, and 15:54 UT.

We also show a zoomed‐in SREM and IES ion penetrating radiation data plot (Figure 4) spanning two days (6 and 7 March 2015/DOY 65 and 66) in order to identify more clearly when the event started. The lower energy electron channel (electron CH2, 0.73 MeV, magenta) starts to show a gradual increase shortly after noon, between the first and second vertical yellow dashed lines. A slight increase can also be observed in electron CH7 (1.35 MeV, dark blue) and the ion penetrating radiation during this interval. The SREM fluxes, including the proton channel (black, 14.5 MeV), and penetrating radiation then all start to increase more steeply from approximately 18:00 UT on 6 March 2015 (DOY 65). They all reach the first major peak just before approximately 06:00 UT, marked by the first green dashed line and therefore also coinciding with Gunell et al. (2018)'s first main infant bow shock crossing. The electron CH2 is a broader double‐peak structure peaking shortly before and after the first green dashed line. The penetrating radiation peak is also broader. An intensity drop is then observed after the first green dashed line for about 4 hr, after which intensities increase again and reach another major peak just after the second green dashed line (10:38 UT). This peak is less prominent in the electron CH2 flux, which generally starts to gradually decrease earlier than the other channels. Slight differences between SREM and the RPC‐IES penetrating radiation may be related to instrument pointing differences. A few more local peaks and troughs are then observed. Although there is some minor variation between channels, the overall trend is the same: The intensity slowly rises to a final prominent, broad peak around 21:00 UT, after which intensities decrease gradually over the following three days and reach previously observed background levels after 9 March 2015 (DOY 68).

#### Discussion of Section 3.1

The decrease in particle flux demonstrated by Rosetta's SREM channel TC2 is likely related to a reduction in cosmic ray particles as a result of an ICME shielding the observation point. Galactic cosmic rays are even higher in energy than SEPs, therefore the fact that only SREM's highest energy channels are affected is in line with this theory. The effect, known as a Forbush decrease, suggests the presence of an ICME in this part of the Solar System, which is ICME *AB*. We further discuss some aspects of this ICME in Sections 3.2.3 and 4. Witasse et al. (2017) describe a similar Forbush decrease detected at Mars, 67P and Saturn, when they tracked an ICME's journey through the Solar System which reached Rosetta on 22 October 2014.

We further discuss specific characteristics and potential sources of this SEP event in Section 4.

### 3.2 Plasma Observations Inside the Comet Coma

In this subsection we present in situ observations of Rosetta's plasma instruments and related calculations in order to investigate the effects of the SEPs on the comet environment.

#### 3.2.1 Cometary Ion Observations

Ions seen mostly at low energies (<10 eV) in the RPC‐IES ion spectrogram in Figure 2c are cometary water group ions. There is also a medium energy (approximately 50–1,000 eV) ion population between ∼19:00 UT on 6 March and ∼06:00 UT on 7 March. This coincides with the onset of the SEP event, and also with a brief spacecraft attitude change, shown in Figure 2e. The comet aspect angle plots indicate that the look direction changed briefly between ∼00:00 and ∼02:45 UT on 7 March, after which it returned to the previous configuration. IES did not see the medium energy population during this period, except for the very last ∼30 min. It then continued to see the population again. We therefore consider this population to be real, but direction‐dependent. To take a closer look at these ions, Figure 2g shows an RPC‐ICA mass‐separated species spectrogram where only cometary ions are plotted. The medium energy population is clearly present in this data set too, indicating that the main composition of this population is water group ions of cometary origin, most likely a mix of H_2_O^+^ and CO_2_^+^; this is probably dominated by H_2_O^+^ at this point in the comet's activity cycle.

##### Discussion of Section 3.2.1

The low energy cometary water group ions (e.g., Goldstein et al., 2015) observed before the event are mostly seen at very low energies (<10 eV) only. This means that they are likely freshly ionized and have not yet been picked up and accelerated by the solar wind flow. However, during the main part of the SEP event (on 6–8 March) we can see a range of energies, up to a few hundred eV.

ICA moment data (not shown) indicate that the ions below 60 eV are flowing radially away from the comet nucleus in the terminator plane which is typical of this population in this region when present (Berčič et al., 2018; Nilsson et al., 2015, 2020). The more energetic ions have a dominating anti‐sunward component, which is also typical of this population here when present. The pickup ions may form partial rings (Nicolaou et al., 2017) but mostly flow along the local electric field on these scales where ions are essentially unmagnetized and electrons magnetized (Alho et al., 2019; Nilsson et al., 2018). Anti‐sunward moving accelerated pickup water group ions can be seen intermittently throughout the mission. However, compared to an overview study of Rosetta ion observations by Nilsson et al. (2017), the observations during this SEP event stand out as a more intense period evident especially in the larger number of ions with energies up to 1 keV during 6–8 March. We note that enhancements can also be caused by stronger electric fields in the solar wind.

The fact that some cometary water group ions are seen at medium energies (approximately 50–1,000 eV) during the SEP event (and therefore at a higher energy than when they are freshly ionized) suggests that they are accelerated pickup ions in a slightly more developed part of their ring distribution, similar to non‐gyrotropic early phase pickup distributions seen by Giotto at comet 26P/Grigg‐Skjellerup (Coates et al., 1993), and also other planetary environments such as Saturn's moons Titan (Regoli et al., 2016), Rhea (Desai et al., 2018; Teolis et al., 2010), and Dione (Nordheim et al., 2020; Tokar et al., 2012) using Cassini observations. 26P/Grigg‐Skjellerup was a generally more active comet when encountered in 1992 than 67P was in March 2015; 67P's neutral production rates were only comparable to 26P's during 67P's most active phase in August 2015 (Coates, 2016; Hansen et al., 2016; Johnstone et al., 1993). Ion gyro radii in 67P's cometary coma environment are very large (probably 1,000s km), therefore we are unlikely to observe fully developed ring distributions with Rosetta (e.g., Nicolaou et al., 2017; Nilsson et al., 2017). Such ions would be expected to have energies a few times higher than the solar wind energy. The medium energy population observed here was probably created 100s km away. The RPC‐ICA pickup ion flux (not shown) falls off with energy in a manner consistent with higher energy ions produced further away from the observation point, in a less dense part of the coma (see e.g., Nilsson et al. (2018), their Figure 4). Local maxima at high energy can also reflect the structure of the comet magnetosphere, and may, for example, be a remote signature of a more developed bow shock, as suggested by Nilsson et al. (2018) and modeled by Alho et al. (2019), Alho et al. (2021).

#### 3.2.2 Solar Wind Ion Observations, Charge Exchange, and Surface Activity

The ions at the higher end of the energy range (around 1 keV and higher) observed in the IES ion spectrogram (Figure 2c) are solar wind ions, as confirmed by RPC‐ICA's mass‐energy spectra (not shown): H^+^ (lower energy band) and alpha particles (He^++^, higher energy band). There is an even higher energy band that becomes visible shortly after the SEP onset (first yellow dashed line) and remains visible for most of 7 and 8 March, with traces also present later on 10 March: these ions are He^+^. The alpha particles observed are usually present in the solar wind, however the He^+^ are not. They are produced as a result of charge exchange reactions between solar wind He^++^ ions and cometary neutrals (Nilsson et al., 2015; Simon Wedlund et al., 2016). The observed count rates of both the cometary and solar wind ion populations increased during the event, especially on 6–8 March. The observed solar wind ions also show slight changes in energy, indicating that the solar wind speed was changing. We explore this further in Figure 3. Figure 3a is the same as Figure 2a, to allow clear orientation and comparison between specific features in Figures 2 and 3. Figure 3b shows an RPC‐ICA mass‐separated species spectrogram showing solar wind ions only, which appear as three bands in the same way as in the IES ion spectrogram: H^+^ (lower energy band), alpha particles (He^++^, higher energy band), and the fainter, intermittently visible highest energy band, He^+^. Figure 3c shows the upstream solar wind speed inferred from Rosetta RPC‐ICA observations of the difference between the speed of protons and alpha particles at the observation point (Nilsson et al., 2022). The data indicate the arrival of high speed streams followed by a linear speed decrease which is a velocity dispersion associated with such streams. This is likely linked to ICME *AB*.

##### Further Analysis and Discussion of Section 3.2.2

In order to investigate how efficient the charge exchange reactions are, we show calculated He^+^ to He^++^ flux ratios in Figure 3d based on RPC‐ICA data. The higher the ratio, the more efficiently the incoming alpha particles have been converted into He^+^ (and eventually He energetic neutral atoms). The calculations are for a 2 AU heliocentric distance approximation and the detailed method is described by Simon Wedlund, Behar, Kallio, et al. (2019), whose Figure 3 also shows a more typical 67P atmosphere for comparison, and with their Figure 4 showing solar wind speed effects on the distributions. The RPC‐ICA He^+^ signal was very variable and sometimes faint, therefore the data shown are somewhat noisy. Nevertheless, Figure 3d indicates an increase in the flux ratio during the SEP event, where the start of the increase coincides with the onset of the SEP event (yellow dashed lines). This increase is approximately a factor of 10 above the level before the arrival of ICME *AB* and SEPs. Large fluctuations in the flux ratios are then present after 8 March which continue another 5–6 days before returning to pre‐event levels (we only show data until the end of 10 March in Figure 3d). At a constant solar wind energy, an increase in this flux ratio is typically a marker for an increased neutral population upstream of the observation point, which may indicate increased surface activity as a result of the SEPs. The presence of ICME *AB* is also likely to have contributed to the increased charge exchange frequency.

Figure 3e shows calculated ionization frequencies as a result of solar wind charge exchange (SWCX), solar wind ionization (SWI, H^+^ and He^++^), and photoionization using RPC‐ICA particle fluxes multiplied by the respective cross sections at the bulk energy of the solar wind species considered (see method described by Simon Wedlund, Behar, Nilsson, et al. (2019) and Simon Wedlund et al. (2020)) with a running average of 6 hr to compensate for factors such as the comet's rotation. The start of the first broad peaks in both the SWCX and SWI appears to coincide with the arrival of ICME *AB*'s shock (blue dashed line) and then reach their highest values during the onset of the SEP event (yellow dashed lines). The SWI peak is slightly more focused on the onset of the main SEP event (second yellow line). It is therefore possible that the increased ionization frequencies are initially caused by the ICME, and then further enhanced by the SEP when the peaks are reached. A second broad increase in both SWCX and SWI is then also present on 8 March. It is unclear why there is a trough between these two peaks. The second peak could be an indirect result of the SEP induced surface sputtering, with Rosetta entering a region where the local neutral population was particularly enhanced and therefore caused increased ionization rates again.

We also investigated neutral number density measurements from Rosetta's Rosina‐COPS instrument (not shown). No clear density enhancements were detected during the event, however high noise levels were present. The data can sometimes be noisy due to slewing, but in this case the degree of noise was considerably higher. The reason for this could be that energetic particles entered the repelling fields inside the COPS instrument and interacted with the emission regulation, a phenomenon that has been reproduced in the lab (pers. comm. with M. Rubin, Tzou, 2017).

#### 3.2.3 Magnetic Field Data and Relation to ICME AB

Figure 2h–2j show RPC‐MAG data in angular coordinates. The data show an overall increase in magnetic field strength during 6–8 March, starting with a fairly sudden increase associated with the arrival of ICME *AB*'s shock (blue dashed line) at 21:25 UT on 5 March. In addition, there is a magnetic field rotation in the elevation angle *θ* on 7 and 8 March as a result of the ICME's flux rope. Specific local magnetic field strength increases can also be observed on 7 March coinciding with the SEP increases and infant bow shock crossings. Specific observations on 7 March, along with proton heating (i.e., a broadening in the H^+^ energy spread) and proton deceleration (i.e., a slight decrease in H^+^ energy) are described in more detail by Gunell et al. (2018).

#### 3.2.4 Electron Measurements, Langmuir Waves, and Surface Charging

An RPC‐IES electron spectrogram is shown in Figure 2b and demonstrates increased count rates during the SEP event on 6–8 March. Some specific increases appear to be associated with the SEP peaks described above, and Gunell et al. (2018)'s infant bow shock crossings (green vertical lines).

The Rosetta RPC‐MIP instrument has two operational modes: active and passive. Active mode is used to monitor electron number densities near the comet while passive mode is used as a natural plasma wave analyzer. Active mode operates in two submodes: Long‐Debye‐Length (LDL) is limited to measuring plasma density below 300 cm^−3^ while Short‐Debye‐Length (SDL) is ideal for measuring densities higher than 300 cm^−3^. On 6 and 8 March, MIP active mode made electron density measurements using LDL submode, while SDL submode was operating on 7 March. The electron density stayed below 300 cm^−3^ on 6 and 8 March for the majority of this time period, therefore RPC‐MIP LDL mode was able to measure electron density. In addition, RPC‐MIP passive mode showed natural wave activity (Langmuir waves) throughout the interval (Figure 11 in Myllys et al. (2021)). As a result the RPC‐MIP passive measurements provided an additional method to estimate the electron density during the SEP event. Based on the combined electron densities from RPC‐MIP active and passive modes, the electron density was enhanced up to 600 cm^−3^ at the end of 6 March and beginning of 7 March (not shown, also see Figure 11 in Myllys et al. (2021)).

##### Discussion of Section 3.2.4

Halekas et al. (2009) showed that SEP events on the Moon can cause extreme surface charging. In addition, Nordheim et al. (2015) modeled surface charging and electrostatic dust acceleration at 67P and found that electrostatic dust ejection from the nucleus can be enhanced for large surface potentials. Dust blow off events in connection to transient solar wind events have also been hypothesized (Flammer et al., 1986). While we do not see any direct evidence of this in the data presented here, it may be of interest to consider for future studies.

The clear enhancement of Langmuir wave activity observed by RPC‐MIP and studied in detail by Myllys et al. (2021) started around 18:00 UT on 6 March 2015; this coincides with the onset of the SEP event. Langmuir waves can be created via a bump‐on‐tail instability when a beam consisting of keV energy electrons is streaming through the plasma. It is therefore likely that the Langmuir wave enhancement is linked to the onset of the SEP event and a signature that SEPs originating at ICME shocks may be effective drivers or triggers of Langmuir waves in cometary environments.

We note that such beams could also be consistent with electron beams from a highly charged surface, as has been observed, for example, at Saturn's moons Rhea and Hyperion (Jones et al., 2011; Nordheim et al., 2014; Roussos et al., 2012; Santolík et al., 2011). At these moons, surface‐originating electron beams were associated with the detection of intense Langmuir wave activity. In the case of the event studied here, this would be consistent with SEPs causing high surface potentials as was observed on the Moon (Halekas et al., 2009).

### 3.3 Discussion

In addition to the discussion points at the end of each of the above subsections, we now briefly describe a few more general considerations. Rosetta was deep inside the comet coma during this event, therefore the responses of any solar transient events are modified by the comet's dense coma. The energetic particles observed at Rosetta resulted in increased ionization rates inside the comet coma, which (at least partially) caused the increase in observed electron and ion fluxes, and electron densities. Ionization rates may have increased as a result of two processes:

1. Energetic particles directly ionizing neutrals in the coma. This could affect the balance between production and loss rates of neutrals. Being at a relatively close distance to the comet nucleus, however, ionization as a loss process is generally expected to be limited; this becomes more important at larger cometary distances (e.g., Heritier et al., 2018) and also depends on heliocentric distance.
2. Energetic particles reaching the comet surface, and striking dust grains, resulting in more surface activity (sputtering). This would increase neutral populations which also leads to more ionization and pickup ions.

In addition, two other processes could have affected the inner coma during the initial part of the event on 6 and 7 March: ICME *AB*'s presence in the vicinity could have compressed the cometary coma, which may have increased the plasma density, and could also have contributed to increased ionization rates through increased electron impact ionization. Furthermore, the observations at the time of the reported infant bow shock crossings include proton heating, sudden increases in magnetic field strength, and an increase in suprathermal electron flux as described in detail by Gunell et al. (2018). As these observed features coincide with the most intense parts of the SEP event it is difficult to discern which observations at these specific times are directly linked to specific processes. The SEP event may have intensified the infant bow shock observations, or enhanced this early stage of the shock formation. The combination of the SEP and ICME may also have moved the cometary infant bow shock closer to the observation point (Rosetta's location), thereby making the signatures clearer. The reconstructed upstream solar wind speed shown in Figure 3c confirms high upstream solar wind speeds reaching above 700 km/s for this time period.

## 4 Upstream of the Comet

### 4.1 Observations of Potential SEP Sources at the Sun

**Figure 5:**
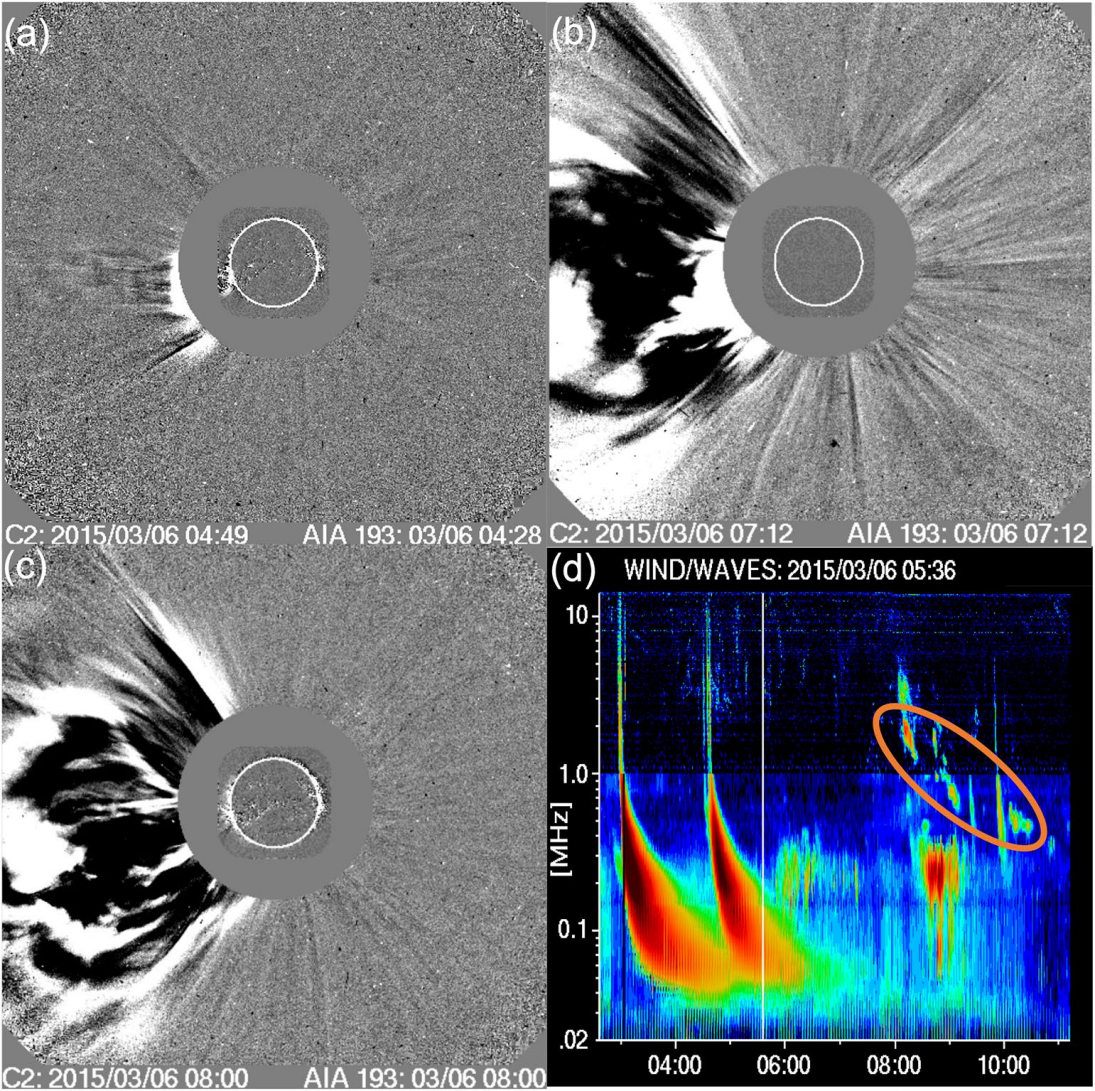
(a) Outer image: SOHO/LASCO C2 difference image showing the first signs of coronal mass ejection (CME) *D* emerging from the Sun at 04:49 UT. Inner image: SDO/AIA 193 difference image at 04:28 UT. (b) Outer image: SOHO/LASCO C2 difference image showing CME *D* and first signs of CME *E* emerging from the Sun on 6 March 2015 at 07:12 UT. (c) Outer image: SOHO/LASCO C2 difference image showing CMEs *D* and *E* emerging from the Sun on 6 March 2015 at 08:00 UT. (d) WIND WAVES radio spectrogram showing frequency on the vertical axis and UT time (on 6 March 2015) on the horizontal axis. The large structures starting at approximately 03:00 and 04:30 UT are two type III radio bursts. The latter was associated with CME *D* and the 4:14 UT flare. The slowly drifting feature just after 08:00 UT marked by the orange circle is a type II radio burst associated with CME *E*. There were no significant type III radio bursts associated with CME *E* and the associated 6:55 UT flare.

A number of CMEs can be seen emerging from the Sun on 6 March throughout the day. Their characteristics are summarized in Table 2, which is an extract from the SOHO/LASCO CME catalog (SOHO/LASCO CME catalog entries: https://cdaw.gsfc.nasa.gov/CME_list/UNIVERSAL/2015_03/univ2015_03.html, Gopalswamy et al., 2009). The table also includes some earlier CMEs; we refer to these later in this manuscript. There were two more recorded CMEs later on 6 March 2015, however they were listed as “very poor events” and are therefore not shown here. The outer image in Figure 5a (SOHO/LASCO image) shows the first signs of CME *D* at 04:49 UT on the Eastern limb of the Sun. CME *E* then follows CME *D* only a few hours later as shown in Figures 5b and 5c where it can be seen together with CME *D*. *E*'s first appearance was recorded at 07:12 UT (Figure 5b). These two CMEs likely interacted with each other and merged because they left the Sun in close succession, and CME *E* was traveling faster than CME *D*.

**Table 2:** Coronal Mass Ejections (CMEs) Observed by SOHO LASCO in Late February–Early March 2015 Which Are Relevant to This Study (Parameters Are From SOHO LASCO CME Catalog (Gopalswamy et al., 2009))

CME	First C2 appearance time (UT)	Central PA (deg)	Angular width (deg)	Linear speed (km/s)	Second‐order speed at final height (km/s)	Notes and references
A	28 February 21:36:05	Halo	360	999	667	*A* starts to merge with *B* at Rosetta's location
B	1 March 17:00:06	303	87	387	392	*A*'s shock arrives at 67P on 5 March
C	3 March 21:38:43	56	56	453	418	
D	6 March 04:38:50	95	155	812	1,103	Associated with 4:14 UT flare and ∼4:30 UT type III radio burst
E	6 March 07:12:05	83	275	880	1,125	Associated with 6:55 UT flare and ∼8:00 UT type II radio burst
						*D* merges with *E* shortly after their eruptions. *DE* studied by MAVEN (Jakosky, Grebowsky, et al., 2015). *DE*'s shock is main source of SEP event
F	6 March 11:12:05	87	77	660	783	
G	6 March 20:48:05	72	79	386	341	

*Note*. The CME labels (A–G) are for the purpose of this study only. C2 refers to the SOHO LASCO C2 coronagraph.

**Figure 6:**
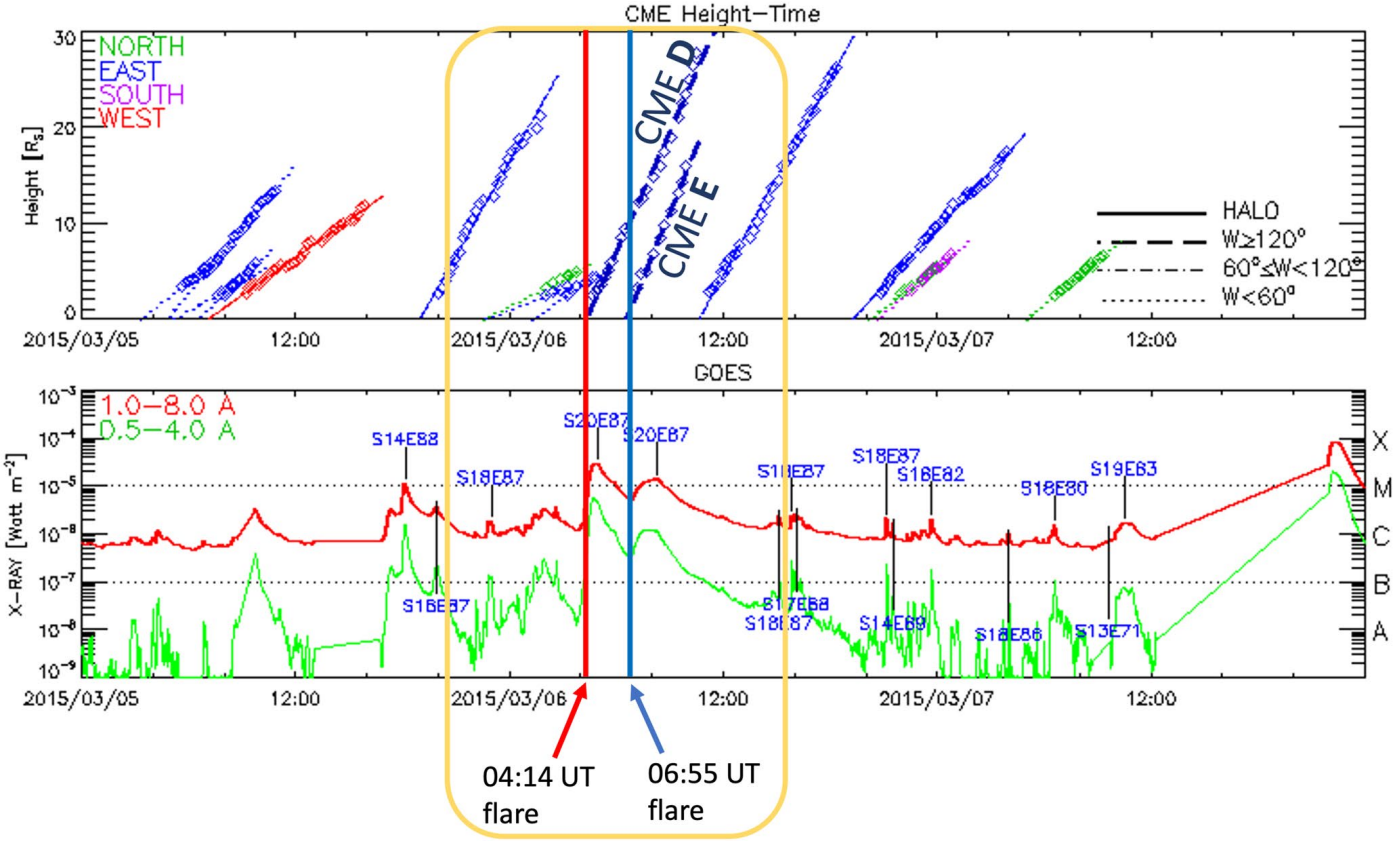
Top panel: SOHO LASCO data showing coronal mass ejection (CME) height including CMEs *D* and *E* on 6 March 2015 highlighted in the yellow box. Bottom panel: GOES SXR data showing two clear flare signatures associated with CMEs *D* and *E*. The flare onset times on 6 March 2015 at 04:14 and 06:55 UT are marked by the red and blue vertical lines, respectively, and indicate how these are linked to CMEs *D* and *E*. Data from Lasco catalog.

According to the National Oceanic and Atmospheric Administration's Space Weather Prediction Center (NOAA SWPC)'s daily event list, which includes all significant flares and their heliocentric coordinates, there were two flares which can be linked to CMEs *D* and *E*, respectively: an M3.0 flare starting at 04:14 UT (4780 0414 0457 0527 G15 5 XRA 1–8A M3.0 9.0E−02 2297) and an M1.5 flare starting at 06:55 UT (4800 0655 0815 0828 G15 5 XRA 1–8A M1.5 6.8E−02 2297). Figure 6 shows how the timing of these flares' X‐ray signatures (bottom panel, GOES SXR data) links to the CMEs (top panel, SOHO/LASCO CME height data). The vertical red and blue lines correspond to the 04:14 UT flare and 06:55 UT flare onset times, respectively, demonstrating that these coincide with the CME onset times at the height‐time extrapolated to one solar radius (*R*_*S*_) due to these being limb events where the projection effects are minimal. The figure also shows that the solar source for CMEs *A* and *B* is the same (S20E87), which is in line with the two CMEs interacting efficiently (Gopalswamy et al., 2004).

The WIND mission's WAVES instrument measures radio disturbances to track radio bursts from Earth orbit. Figure 5d (created using the NASA CDAW service: https://cdaw.gsfc.nasa.gov/movie/make_javamovie.php?img1=lasc2rdf%26img2=wwaves%26date=20150306) shows two large structures, starting at ∼03:00 and ∼04:30 UT, which are visible at almost all frequencies but broader at the lower end. These are type III radio bursts and the latter is linked to the 04:14 UT flare and CME *D*. The WIND WAVES observations also show a type II radio burst just after 08:00 UT (highlighted by the orange circle), which is close in time to CME *E*'s first appearance at 7:12 UT. Such bursts are generated by accelerated electrons at shocks, which in this case is evidence for the presence of a shock front driven by CME *E*; this is crucial information in terms of determining the source of the SEP event. We note that type II bursts typically occur together with type III bursts, however this is not always the case because open field lines are not always present to carry the flare accelerated electrons. In this case no type III radio bursts were observed that could be associated with CME *E*. We also note that the frequency range of the type II burst indicates the distance to the Sun was only a few solar radii, therefore this likely occurred before CME *E* merged with CME *D*. No other type II radio bursts were observed on 6 March, indicating that there is no direct evidence for shocks driven by any of the other CMEs.

### 4.2 ENLIL Simulation

**Figure 7:**
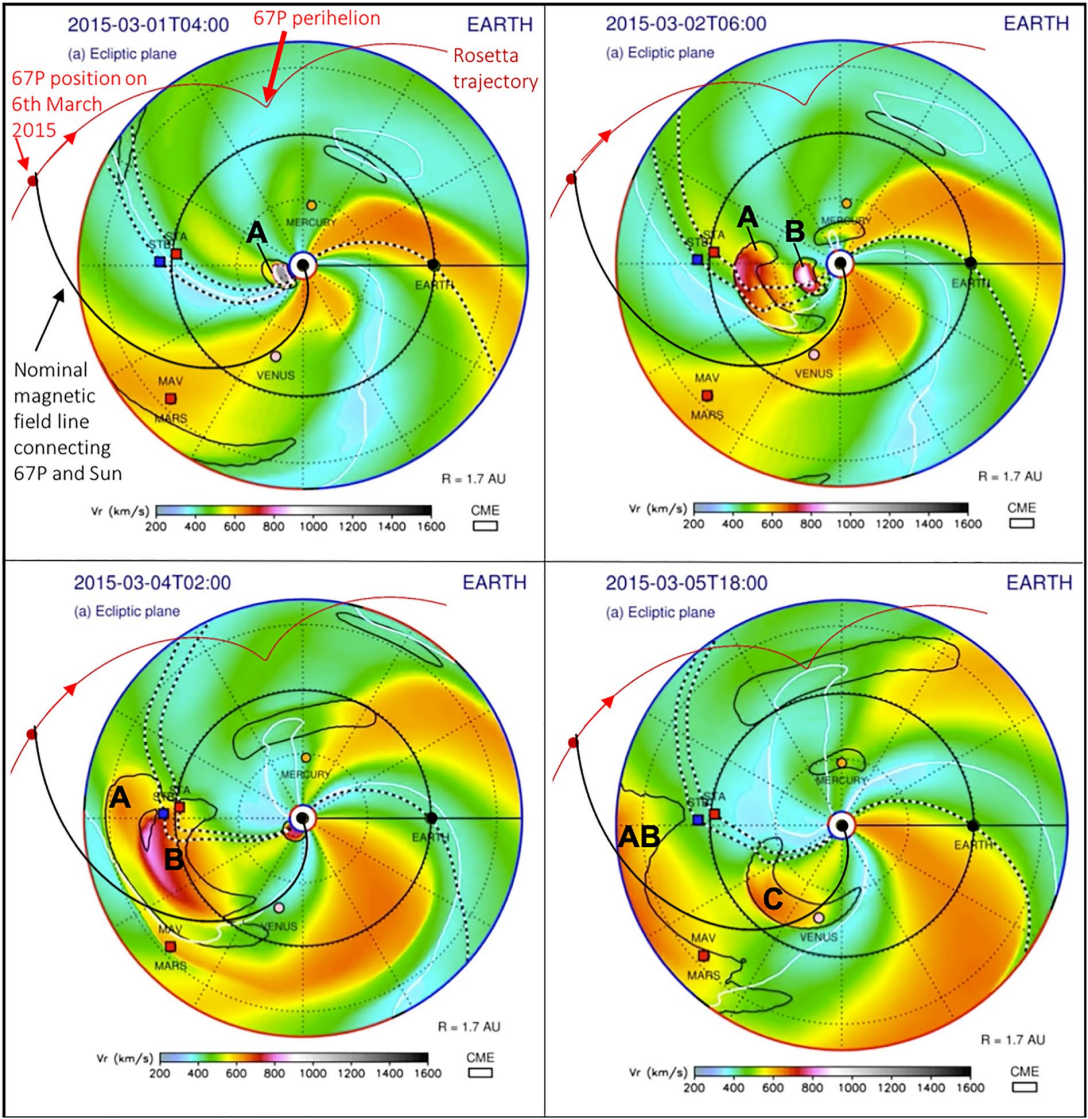
ENLIL simulations of the inner Solar System Part 1: 1–5 March 2015. The Sun is in the center and the color scale indicates simulated solar wind velocities. Date and time are shown in the top left corner of each panel, in UT. The STEREO spacecraft (STA and STB) are shown, as well as major interplanetary coronal mass ejections (ICMEs) (structures surrounded by black solid curved lines). Parker Spiral magnetic field lines that connected the Sun and the STEREO spacecraft, and the Sun and Earth, are shown as black and white lines. The added red line indicates the trajectory of comet 67P, including its perihelion position, its position on 6 March 2015 and the direction toward perihelion. An additional magnetic field line has been added (spiral black solid line) to demonstrate a nominal Parker Spiral field line crossing the location of the comet. Some ICMEs relevant to this study are marked using capital letters; their characteristics are summarized in Table 2.

**Figure 8:**
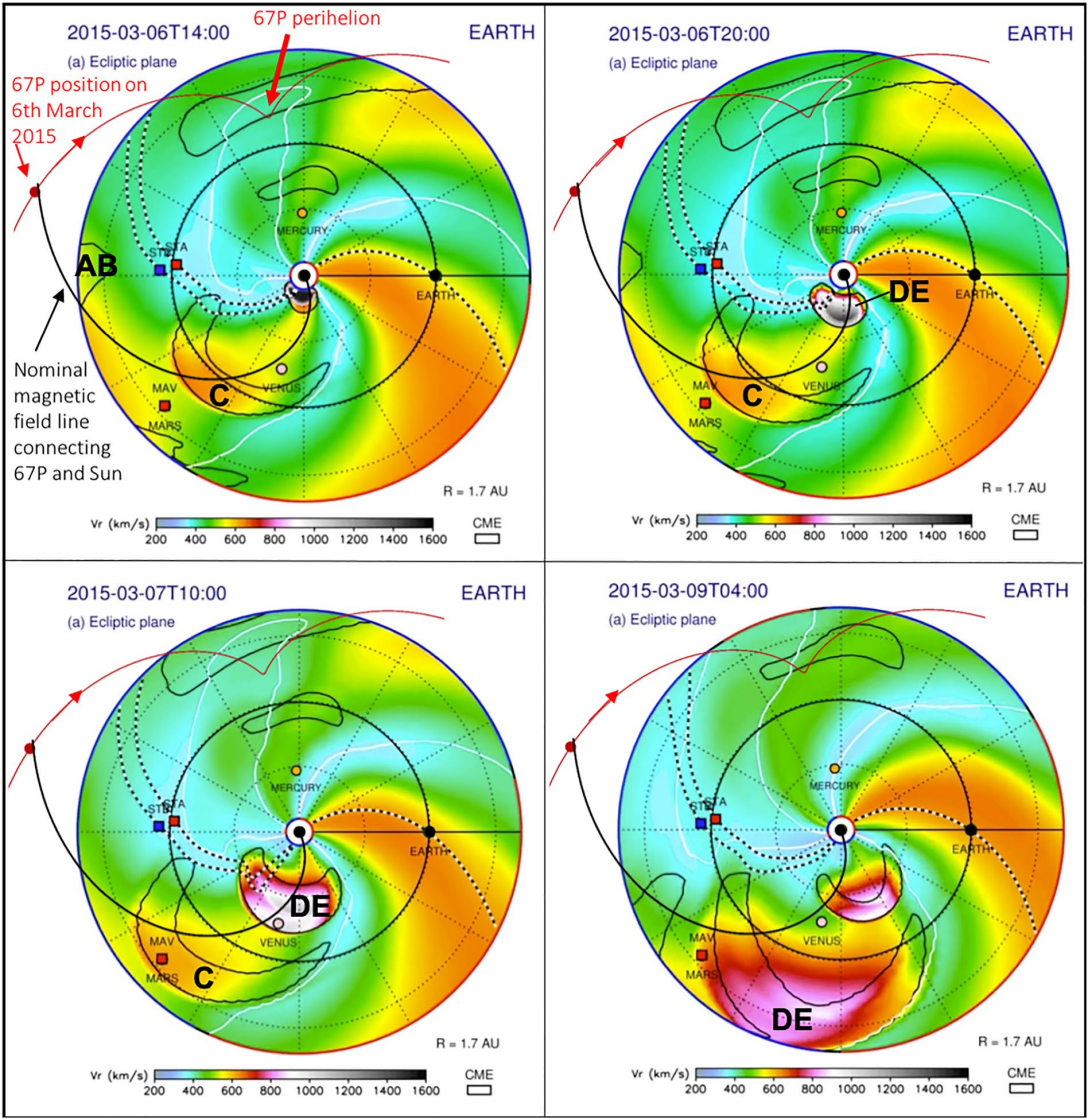
ENLIL simulations of the inner Solar System Part 2: 6–9 March 2015. The Sun is in the center and the color scale indicates simulated solar wind velocities. Date and time are shown in the top left corner of each panel, in UT. The STEREO spacecraft (STA and STB) are shown, as well as major interplanetary coronal mass ejections (ICMEs) (structures surrounded by black solid curved lines). Parker Spiral magnetic field lines that connected the Sun and the STEREO spacecraft, and the Sun and Earth, are shown as black and white lines. The added red line indicates the trajectory of comet 67P, including its perihelion position, its position on 6 March 2015 and the direction toward perihelion. An additional magnetic field line has been added (spiral black solid line) to demonstrate a nominal Parker Spiral field line crossing the location of the comet. Some ICMEs relevant to this study are marked using capital letters; their characteristics are summarized in Table 2.

ENLIL (Odstrcil, 2003) is a large scale, physics based prediction model of the heliosphere used by the Space Weather Forecast Office (http://helioweather.net/). Figures 7 and 8 show plots from an ENLIL simulation during 1–9 March 2015, including a number of prominent ICMEs during this period. The full video clip is also available (http://helioweather.net/archive/2015/03/. Also see ENLIL run https://ccmc.gsfc.nasa.gov/database_SH/Leila_Mays_122116_SH_2.php). The area shown is the inner heliosphere, with the inner planets and also the STEREO spacecraft (STA and STB). The color scale indicates simulated solar wind velocities, and the date and time is shown in the top left corner of each panel.

The ICMEs labeled *A* and *B* can be seen emerging from the Sun on 1 March 2015. ICME *B* probably caught up with ICME *A* around 1 AU from the Sun and they started to interact and/or merge near Rosetta's location. ICMEs *A* and *B* encountered the STEREO A spacecraft in close succession on 2 and 3 March, respectively (data not shown here, the detailed study of these two ICMEs is the focus of a future study in preparation). ICME *C* emerged from the Sun on 3/4 March but traveled in a different direction. ICMEs *D* and *E* (Figure 8) appear as one large, merged ICME and can first be seen between 04:00 and 06:00 UT on 6 March. They correspond to the CMEs shown in Figure 5. The merged ICME *DE* can then be seen continuing to travel toward Venus; it never encountered the STEREO A and Rosetta spacecraft. ICMEs *F* and *G* do not appear in this simulation due to their smaller size and lower speed.

67P encountered ICME *AB*'s Eastern flank on 5/6 March2015: ICME *AB*'s shock arrived at Rosetta on 5 March at 21:25 UT (vertical blue dashed line in Figures 2, 3, 4). In addition, Jakosky, Grebowsky, et al. (2015) describe the interaction of an ICME with the Martian atmosphere using MAVEN data (also see Curry et al., 2015; Duru et al., 2017). They report that the ICME reached Mars on 8 March 2015 and identified it as the merged ICME we refer to here as *DE*. It therefore arrived at Mars slightly earlier than the predicted date (the morning of 9 March 2015) by the ENLIL model. However, the model results show that only the Eastern flank (i.e., the left part in this figure) of the ICME encountered Mars, and that the central part passed the Martian orbit on 8 March. It is therefore possible that the timing is actually accurate, but the modeled direction of the ICME is slightly off and the central part of the ICME did encounter Mars directly. This appears to be in line with the observed substantial effects on Mars as described by Jakosky, Grebowsky, et al. (2015).

The black and white lines show Parker Spiral magnetic field lines that connect the Sun and the STEREO spacecraft, and the Sun and Earth. We added red lines showing the path of 67P in the heliosphere, including its location on 6 March 2015 when Rosetta was 2.15 AU from the Sun. We also added a nominal Parker Spiral magnetic field line (black solid line) that crossed the location of the comet on 6 March to demonstrate what the comet may have been magnetically connected to in addition to the Sun, such as the leading edge of ICME *DE*. At a solar wind speed of 400 km s^−1^, the path length of this spiral is 3.42 AU. The actual magnetic field lines connecting, for example, ICME *DE* and Rosetta, would be disturbed by the ICME, and would not be a neat spiral as shown, however this is difficult to model accurately.

### 4.3 Observations of SEP Event at STEREO A and Mars

**Figure 9:**
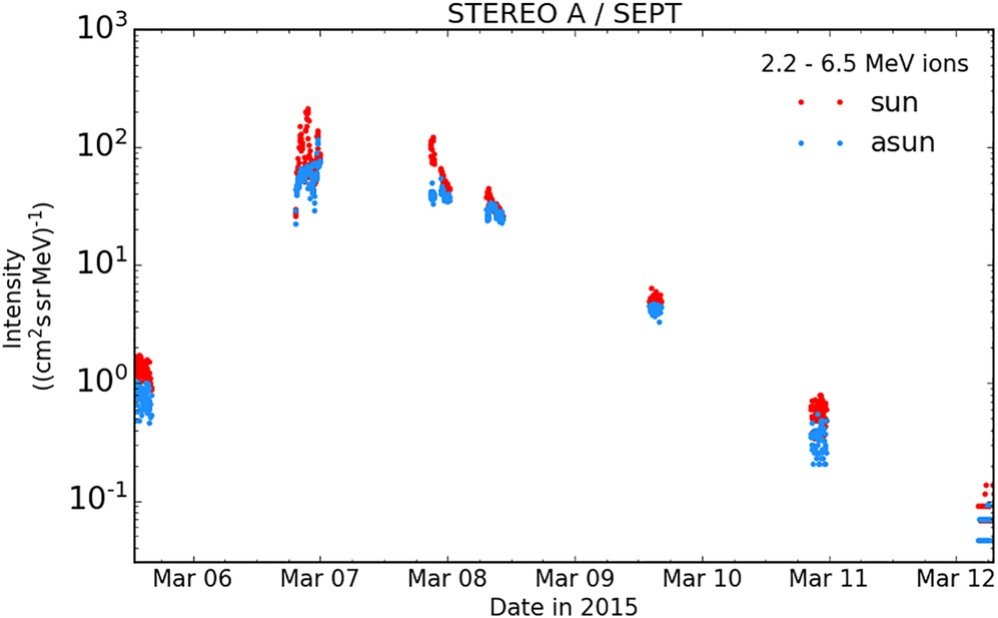
Solar Terrestrial Relations Observatory Ahead (STEREO A) Solar Electron and Proton Telescope (SEPT) data showing an increase in solar energetic particle ion flux during 6–11 March 2015. Directional information shows red data points coming from a sunward direction and blue data points coming from an anti‐sunward direction.

**Figure 10:**
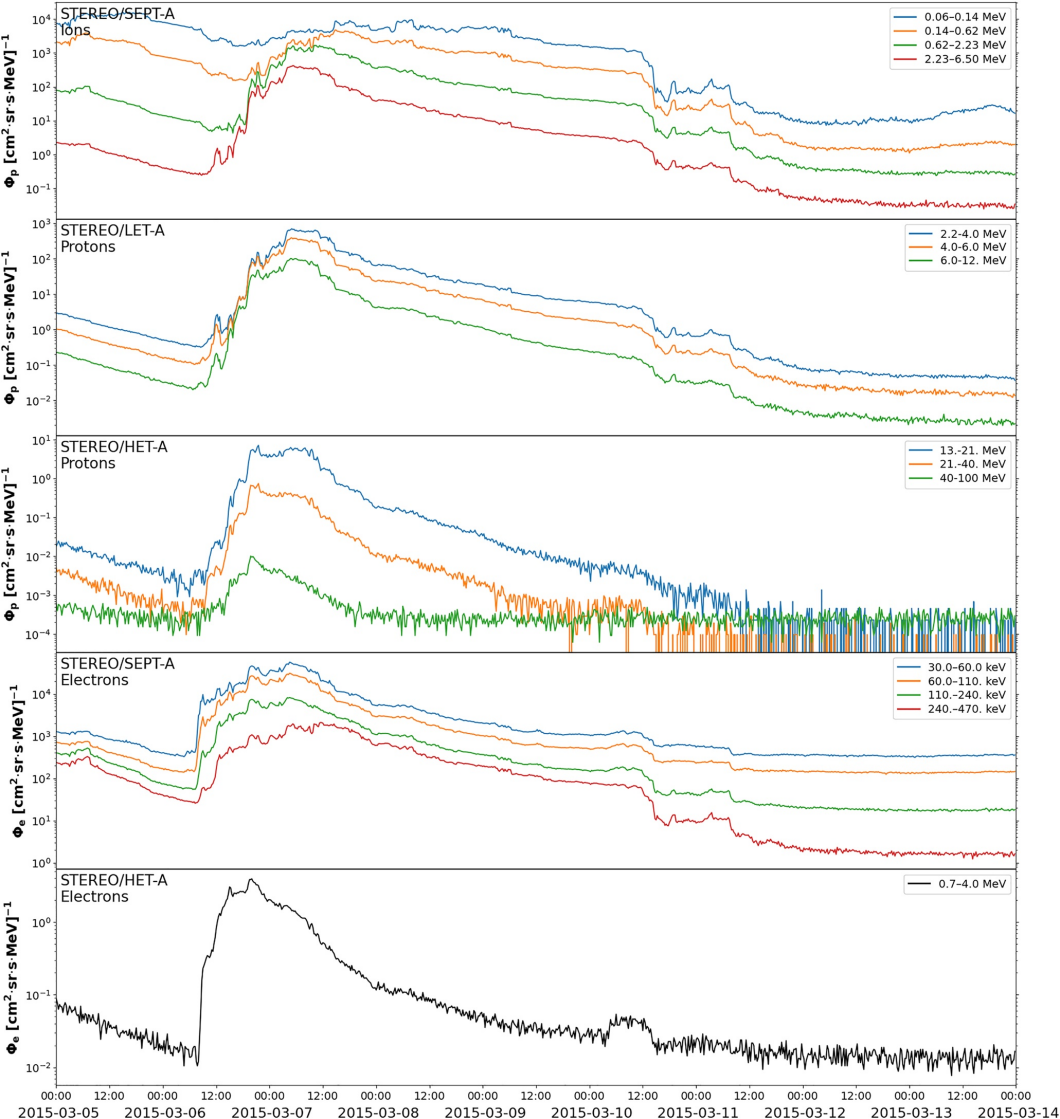
Solar Terrestrial Relations Observatory Ahead (STEREO A) Solar Electron and Proton Telescope (SEPT), Low Energy Telescope (LET), and High Energy Telescope (HET) flux‐time profiles of the solar energetic particle event during 5–14 March. The top, middle, and bottom panels show ion, proton, and electron data, respectively as indicated. The colors correspond to different energy channels as shown. Note these are lower rate beacon telemetry mode data.

The position of the STEREO A spacecraft is indicated in Figures 7 and 8 as STA. Energetic particle observations from the SEPT instrument are shown in Figure 9. The data range spans 5–12 March 2015 and the energy channel shown is 2,224–6,500 keV ions coming from sunward directions (red) and anti sunward directions (blue). The measurements contain several data gaps because of the greatly reduced telemetry rates during STEREO's superior conjunction where their high gain antennas had to point at an angle a few degrees away from Earth. A clear and significant increase in ion intensity can still be observed starting on 6 March 2015 (DOY 65). The intensity then gradually returns to previous levels by 11 March 2015 (DOY 69). The directional information also shows an anisotropic distribution, with a higher intensity of protons coming from a sunward direction. The timing and duration of this event therefore suggest that the source is likely the same as that of the SEP event observed at Rosetta. Figure 10 shows SEP flux‐time profiles of this event from the STEREO A LET, HET, and SEPT instruments. Note that these are beacon data which is a low rate, highly compressed telemetry data mode. We further characterize this event at the end of Section 4.4.

Jakosky, Grebowsky, et al. (2015)'s study of ICME *DE* also discusses observations of SEPs using MAVEN's SEP instrument. Figure 1 in Jakosky, Grebowsky, et al. (2015) shows that MAVEN initially observed a moderate increase in low energy SEPs (mostly 10s of keV) starting in the afternoon of 6 March, after which a major >1 MeV SEP event (marked *E*3 in their figure) reached Mars at 08:00 UT on 7 March. This is well ahead of the main ICME *DE* disturbance which arrived at 15:20 UT on 8 March (marked *S*4 in their figure), suggesting that these SEPs traveled to Mars, most likely via magnetic field lines, from the production source. The source in this case is likely to be a combination of the two flares, and the ICME shock front, as discussed further below.

**Figure 11:**
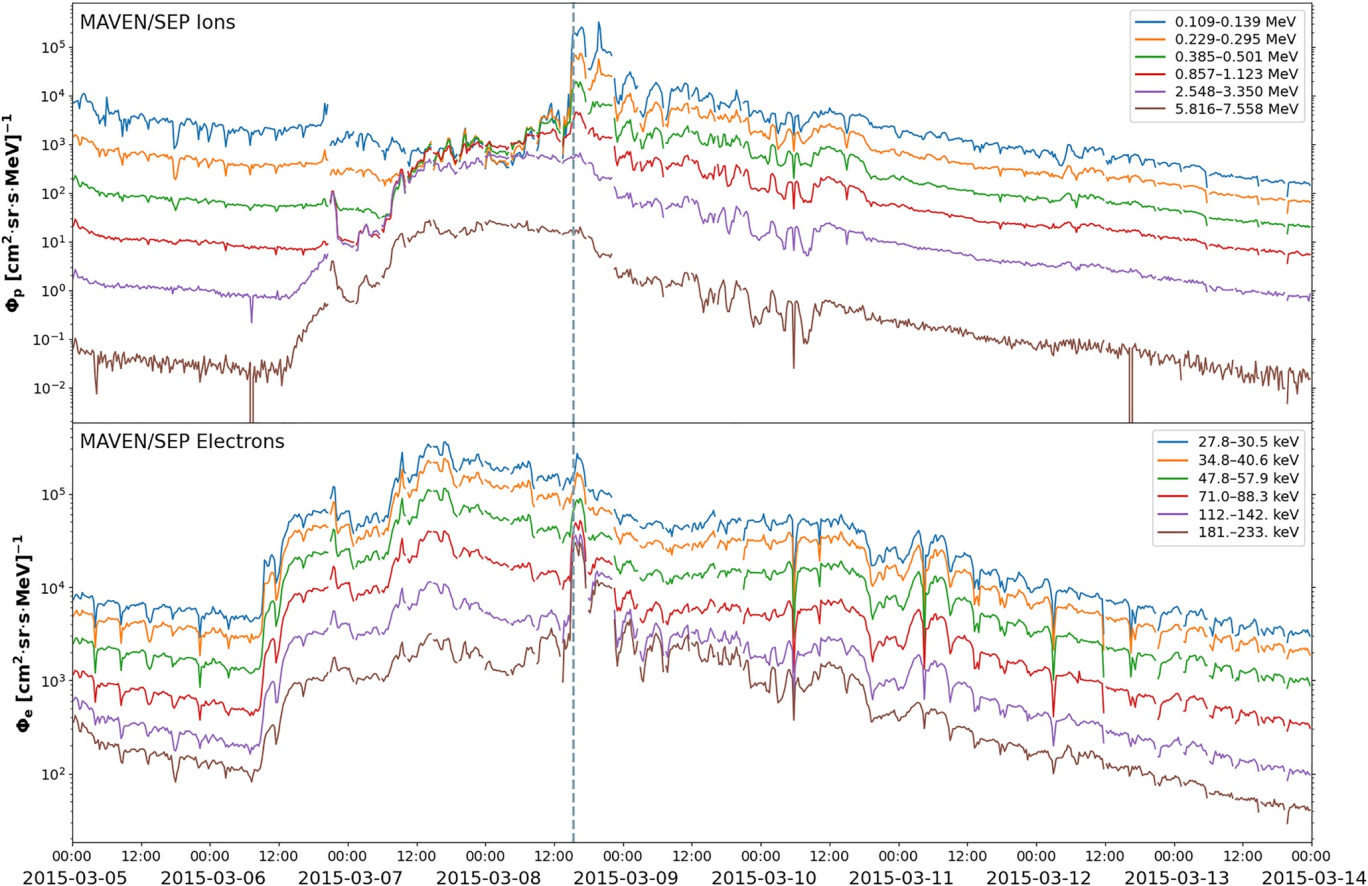
Mars Atmosphere and Volatile Evolution‐Solar Energetic Particle (MAVEN‐SEP) data of selected energy channels as indicated by the colors showing ion (top panel) and electron (bottom panel) flux‐time profiles of the SEP event at Mars. The vertical dashed line (15:20 UT on 8 March) marks the arrival of interplanetary coronal mass ejection *DE*'s shock at MAVEN.

At the time of ICME *DE*'s arrival at MAVEN, a surge of SEPs were detected for a few hours, covering an energy range of 10s keV to a few MeV. These were directly associated with the ICME's shock accelerating particles locally. After the passage of the main front, the MAVEN SEP instrument continued to observe 10–100s keV energy ion populations and 10s keV energy electrons until approximately the end of 9 March during the passage of the remaining parts of the ICME. The intensity of these particles gradually decreased during this time period. Further observations of 10s keV ions and electrons on and after 9 March are likely associated with another flare on this date as marked in Jakosky, Grebowsky, et al. (2015)'s Figure 1. We show MAVEN SEP flux‐time profiles from a selection of energy channels of this event in Figure 11. The vertical dashed line indicates when ICME *DE*'s shock front arrived at MAVEN. We further discuss these profiles at the end of Section 4.4.

**Figure 12:**
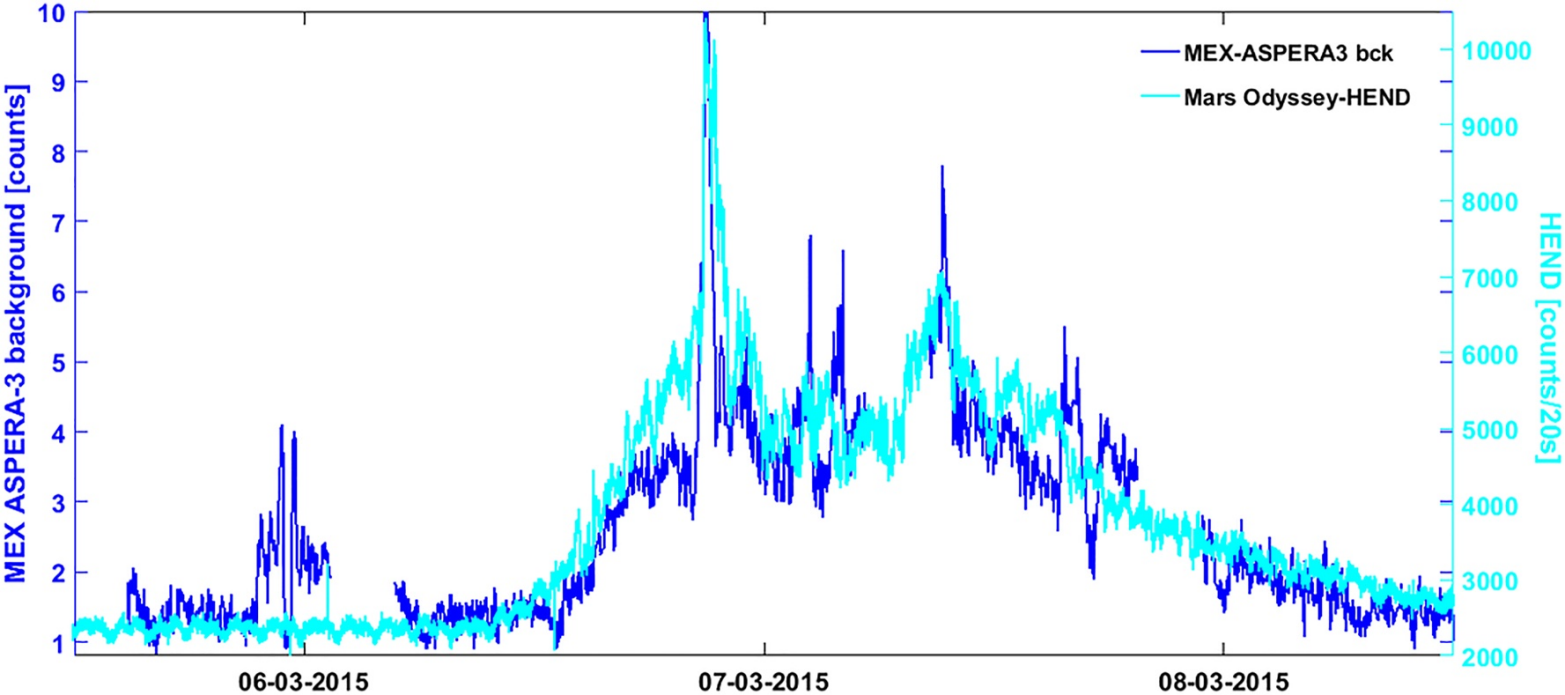
Normalized Mars Odyssey High Energy Neutron Detector (HEND, light blue) and Mars Express (MEX) Analyzer of Space Plasma and Energetic Atoms Ion Mass Analyzer (ASPERA‐3) penetrating radiation (dark blue) data during the evening of 5 March 2015–evening of 8 March 2015. Both data sets are sensitive to energetic particles, mostly in the 100s keV+ energy range, and show a solar energetic particle event during approximately 6 March (afternoon)–8 March (evening), that is, before and during the passage of major interplanetary coronal mass ejection *DE*.

Figure 12 shows normalized Mars Odyssey HEND data and Mars Express ASPERA‐3 IMA penetrating radiation spanning 3 days from the evening of 5 March 2015 to the evening of 8 March 2015. The penetrating radiation shown is the background counts recorded in the highest energy channels of the IMA sensor, similar to the way we used specific RPC‐IES energy channels to study the penetrating radiation at Rosetta. These IMA background counts are the result of multiple factors, such as the impact of high energy particles, ultraviolet photon fluxes, electronic effects, data processing errors, or other unknown effects. In our case, since we are studying the propagation of an SEP, energetic particles penetrating the instrument housing is the most probable scenario. Although we do not know the precise nature of the particles observed by HEND and the IMA penetrating radiation, and whether they are exactly the same types of particles, previous studies have shown that both instruments correlate very well during space weather events at Mars (e.g., Jiggens et al., 2019; Morgan et al., 2014). The correlation between the data sets shown in Figure 12 is high and demonstrates a significant increase in SEP intensity from approximately the afternoon of 6 March until the evening of 8 March. These observations therefore start slightly earlier than the MAVEN SEP observations, which is likely due to the instrument limitations as described above, and slightly different energy ranges. Nevertheless, MAVEN SEP, HEND, and Mars Express IMA observations generally agree well in that a significant energetic particle event was observed at Mars starting on 6 March, that is, well before the arrival of ICME *DE*, which was likely the main source of the SEP event.

### 4.4 SEP Source Discussion

Based on the observations and simulations presented we now consider and discuss possible sources of the SEP event observed by Rosetta. The energetic particles observed by Rosetta's SREM instrument are highly unlikely to have been produced locally due to the lack of a local event that could have accelerated particles to such high energies. Gunell et al. (2018) describe Rosetta RPC observations on 7 March 2015 associated with the formation of an infant bow shock. Earlier studies of comet encounters in the Giotto era (e.g., Galeev, 1991) showed that even comets with fully developed bow shocks did not produce particles in excess of 500 keV. Therefore any infant bow shocks present on 7 March 2015 are highly unlikely to have been the source of protons with energies >12 MeV and electrons with energies >0.65 MeV observed here. It is possible that some particles were accelerated to lower energies, however it is more likely that the presence of the SEP event observed, together with ICME *AB*, enhanced the formation of these infant bow shock signatures, as discussed in Section 3.

Part of ICME *AB* (most likely its Eastern flank as described above) encountered the 67P environment on 5/6 March and may have contributed to some particle acceleration and some of the observed magnetic field observations. Myllys et al. (2021) report the ICME's shock arriving at 21:25 UT on 5 March, which is in line with RPC‐MAG observations shown above. In cases where a CME is the SEP source, the SEPs are produced at the CME shock. Therefore the SEPs can be observed before the arrival of the CME shock (because they travel much faster than the CME itself), and during the arrival of the shock, but not afterward. We cannot see any enhancement in the SREM or IES penetrating radiation data on 5 March. The main SEP event onset observed in this study took place over one whole day after the arrival of the shock, and lasted until the end of 9 March. It is therefore unlikely that ICME *AB* was the source of this SEP event. Furthermore, the SEP event signatures observed at STEREO A and Mars would not be associated with a production mechanism local to Rosetta due to the lack of direct magnetic connections between these locations. The events at STEREO A and Mars took place on and after 6 March, at which time ICME *AB* had already reached much larger distances from the Sun than STEREO A and Mars.

We therefore deduce that the production source of the SEPs is a remote location with a magnetic connection to all SEP event observation sites at Rosetta, STEREO, and Mars. CME *C* is unlikely to have been associated with any shocks intense enough to accelerate particles to the observed energies considering its modeled size and velocity, and also the lack of any type II radio burst observation in Wind Waves data that could be associated with it. According to the ENLIL simulations it is also unlikely to have had a magnetic connection to STEREO after the end of 6 March 2015, which is when the SEP event was only starting. Nevertheless, it is possible that CME *C* may have contributed to some of the observed SEP counts.

The 4:14 UT solar flare described above is likely to be directly or indirectly linked to the production of the SEPs observed at Rosetta, STEREO A, and Mars. The ENLIL simulations demonstrate that nominally, magnetic connections were present between the side of the Sun where the flare occurred and the observation sites at Rosetta, STEREO A and Mars. In addition, the timing of the SEP onsets at STEREO A and Rosetta is consistent with the flare time stamp and travel times of energetic particles via Parker field lines.

However, the actual SEP event observations at Rosetta, STEREO A, and Mars appear to also share characteristics with the gradual event category (associated with production at CME shocks). The prolonged duration of the event together with the observed proton anisotropy at STEREO A is a strong indicator that the flare is unlikely to be the main source of the particles for the entire event; flares typically only produce SEPs for minutes up to a few hours. Acceleration areas of flare sites on the Sun are also generally small and therefore accelerated particles propagate out to a relatively narrow spread in the heliosphere because only a limited number of magnetic field lines are connected to the small acceleration region. Such magnetic field lines are therefore not likely to connect to a broader spread in the interplanetary medium. Rosetta and STEREO A were reasonably well aligned radially, but not “magnetically”, that is, their magnetic connections to the Sun were via different magnetic field lines. Mars, on the other hand, was further apart from Rosetta and STEREO in terms of longitudinal separation as shown in the ENLIL figures, but closer in magnetic field alignment with Rosetta. We note that SEPs can also scatter across field lines, however the fact that the observations at STEREO A are anisotropic suggest that this is not the case for the majority of SEPs detected. The most likely candidate for an additional main source of the SEPs is the merged CME *DE*. Based on our analysis above, the original CME *E* is associated with the type II radio burst shown in Figure 5d which infers the presence of a shock front associated with this CME. Jakosky, Grebowsky, et al. (2015)'s observations of ICME *DE* using MAVEN data at Mars also confirm the presence of a shock producing energetic particles. ICME *DE*'s shock front is consistent with the production of particles reaching the observed energies. It is also consistent with the more gradual nature of the SEP events observed, and the longer duration; the ENLIL simulations demonstrate that magnetic connections between the front of ICME *DE* and the SEP observations sites are nominally present for the duration of the events, and also likely even if this particular ENLIL run is not fully accurate. Therefore the shock could have continuously accelerated particles which then traveled to the observation sites. The relatively large angular size of the shock front creates a much larger acceleration region to which magnetic field lines can be connected and therefore allow energetic particles to propagate out to a broader spread in the interplanetary medium. This can therefore include Mars before the arrival of the ICME there, but also the STEREO A and Rosetta observations sites, without this ICME ever being in their immediate vicinity.

Furthermore, the fact that ICMEs *D* and *E* interacted and very likely merged may have enhanced the shock (Dresing et al., 2018) which was originally driven by CME *E*. This type of interaction between ICMEs can also lead to an intensification of particle acceleration (Li et al., 2012), which is in line with seeing relatively high energy particles being produced for such a prolonged period of time, and Jakosky, Grebowsky, et al. (2015)'s description of this ICME being “one of the strongest ever observed at Mars.” In addition, ICMEs elsewhere in the interplanetary medium can have a strong influence on SEP transport such as facilitating the spread of particles over broad longitudes (Palmerio et al., 2021). Considering ICME *AB*'s trajectory and encounter with 67P, it is likely that its presence promoted the transport of SEPs to Rosetta from ICME *DE* and enhanced particle fluxes.

The ENLIL model run we presented gives a general idea where magnetic connections might be present, and approximate locations of the ICMEs. We have also considered other ENLIL runs where ICME trajectories turn out to be very similar, and with magnetic connections that support our theory outlined above. We note that although these runs are based on magnetograms and Potential Field Source Surface extrapolations (that is, they are observation based), the CME variables are entered manually and therefore strongly depend on the available data. Despite the limitations of these simulations, they strongly support our theory because even if the ICME trajectories are slightly different, or their speeds and therefore the timings slightly different, clear magnetic connections between *DE*'s shock and the SEP observations are still very likely and therefore CME *DE* together with the flares on 6 March are still the most likely sources to have produced the observed SEP events.

SREM fluxes show that the electron SEP onset time at Rosetta is a few hours earlier for electrons than for protons. This is consistent with electrons traveling faster along field lines: For electrons and ions that are produced at the same time and location at the CME shock, electrons will arrive at the observation point earlier than the ions. The electron and ion penetrating radiation also showed a slight increase in intensity above the background level before the SREM proton onset; the reason for this in unclear, however it may be related to instrumental effects such as different pointing. Lower energy observations are also linked to higher intensities and are therefore easier to detect above background levels, which also vary for different channels. In addition, it is possible that some of the electrons and protons are injected at different locations at the shock.

The variability observed in the SEP events (such as the initial peak followed by a local trough and then a higher intensity peak) can be a signature of slight changes in magnetic connectivity and further points toward a broader source region such as an ICME. The magnetic connection allowing energetic particles to travel from the source is constantly in motion and can therefore move from a region where particle acceleration is efficient and intense to, for example, where it is more moderate or even completely absent. This can also happen more readily where the source region is dynamic and broad, such as an ICME shock.

Another reason for the variability could be the presence of flux tubes in the interplanetary medium with varying scattering conditions. Flux tubes with more scattering will cause particle fluxes to be more evenly distributed along field lines. When a remote observer enters such a flux tube, a drop in particle intensity can be observed. In order to disentangle the precise reasons, directional detection capabilities to check for local anisotropies would be required at Rosetta. Similarly, the slight differences in timing and energy of the observed SEP event at Mars prior to the arrival of the ICME by MAVEN SEP, Mars Odyssey HEND, and Mars Express ASPERA‐3's IMA penetrating radiation, in addition to instrument limitations and differences in energy coverage as discussed above, may also be related to varying degrees of the magnetic connectivity to ICME *DE*'s shock and possibly the flares as a result of being in physically different Martian orbital locations.

We can explore some more basic characteristics of the SEP event by comparing the intensity‐time profiles at different heliocentric longitudes. In order to do this we need to compare data in the same energy range. The STEREO A LET and MAVEN SEP instruments have some channels whose energy ranges overlap, such as the 6–12 MeV STEREO A LET proton channel (Figure 10, second panel, green trace), and the 5.8–7.6 MeV MAVEN SEP ion channel (Figure 11, top panel, gray/brown trace). The onset time and duration (approximately 5–6 days) of these profiles look similar. Considering the relatively large longitudinal separation between STEREO A and MAVEN, this is somewhat unexpected. Typically, SEP events with longitudinal separations on this scale tend to have different profiles depending on how well the observation point is connected to the shock front, and to which part of the shock front the observation point is connected, as described by, for example, Lario and Simnett (2004). These relatively similar profiles suggest that both STEREO A and MAVEN had reasonably good magnetic connections to ICME *DE*'s shock and that the particle production mechanisms locally at the shock were similar. However, despite the fact that MAVEN actually encountered ICME *DE*, the SEP rise phase observed at STEREO A appears to be more intense and with a higher peak flux of 102 cm^2^ sr s MeV^−1^ compared to 27 cm^2^ sr s MeV^−1^ at MAVEN. Note that the energy ranges of these channels are not an exact match, with the STEREO A LET channel covering a larger range. Nevertheless, these points indicate that STEREO A may have had a connection to a more efficient particle acceleration site at the shock front such as ICME *DE*'s nose. This is roughly in agreement with the ENLIL model shown above, which indicates that a connection between STEREO A and the nose of ICME *DE* would have been possible when ICME *DE* was still relatively close to the Sun (see the two top panels of Figure 8 on 6 March with UT times as shown). At this relatively early time in the ICME's life cycle the particle acceleration mechanisms at the shock front would have also been at a more efficient stage, which is also in line with the more intense rise phase observed at STEREO A. MAVEN, on the other hand, may have been connected to the nose of ICME *DE* at a slightly later stage of ICME *DE*'s life cycle, such as the bottom left panel of Figure 8 on the morning of 7 March. This would also explain the slightly prolonged maximum intensity phase observed at MAVEN from just before midday on 7 March until the arrival of the ICME in the afternoon of 8 March.

Unlike the higher energy ion channels, MAVEN SEP's lower energy ion channels (approximately <1.1 MeV) do not show a significant intensity increase before the arrival of the shock. This may indicate that a prior structure in the solar wind, or perhaps even in the Martian ionosphere, prevented these ions from reaching MAVEN remotely via magnetic field lines. They were then detected as the production source (i.e., the shock) arrived at MAVEN and produced these SEPs locally. This structure does not appear to be present at STEREO A: Similar SEPT energy channels (Figure 10, top panel, blue, yellow, and green traces) do show elevated intensities throughout the event compared to the background levels afterward. However, the lower energy channels do not show very sharp rise phases, especially at the lowest energies observed. We note that SEP intensities at STEREO A just before the SEP event discussed here were still slightly above nominal background levels due to the passage of ICMEs *A* and *B* during 2–4 March. The presence of ICMEs *A*, *B*, and *C* may have had an effect on SEP transport from ICME *DE*. This could include a particle reservoir effect (e.g., Y. Wang et al. (2021), Anastasiadis et al. (2019), and references therein) in the inner heliosphere which may also have contributed to the prolonged duration of the SEP event at STEREO A and Mars.

We can also compare some basic characteristics of selected STEREO A HET and Rosetta SREM channels that have some overlapping energy ranges. The shape of STEREO A's HET 13–21 MeV proton intensity‐time profile (blue trace, middle panel of Figure 10), and Rosetta's SREM 14.5 MeV proton profile (black trace, Figures 2, 3, 4) is similar with a similar onset time; the STEREO onset time is approximately 2 hr earlier than Rosetta, in line with STEREO A being closer to the source. This suggests that STEREO A and Rosetta may have been connected to similar proton production locations at ICME *DE*'s shock front. It is also possible that the magnetic field lines connecting Rosetta and ICME *DE*'s shock were moved closer to STEREO as a result of ICME *C'*s presence. In Figure 8, an example of this would be that the nominal field line connecting Rosetta and ICME *DE* would be bent around ICME *C'*s Eastern flank, that is, toward STEREO A's location/the top of the page. A slightly different ENLIL run (http://helioweather.net/) demonstrates such a scenario. Some differences between these STEREO A HET and SREM proton profiles include a slightly longer duration at STEREO A where the event lasted about 5 days which is over a day longer than at Rosetta, indicating that Rosetta's connection to the shock lasted a shorter period of time. We can also see a significantly higher peak intensity at STEREO A, but note that the energy channels discussed here do not have closely matching energy ranges with the 13–21 MeV HET channel covering a larger range than the 14.5 MeV SREM channel. The higher peak intensity may indicate that STEREO A was connected to a more efficient proton production site at the shock location such as the ICME's nose.

STEREO A's HET 0.7–4.0 MeV electron channel shown in the bottom panel of Figure 10 also overlaps with the Rosetta SREM electron channels shown in Figures 2, 3, 4 (magenta and blue traces). The early rise phase at Rosetta is more gradual than at STEREO A, therefore STEREO A likely had a better connection to an electron production site at the shock at this point. After these initial differences, the rest of these channels' profile shapes are similar. The SREM CH2 peak intensity is higher, however the overall intensity increase is just under 1 order of magnitude which is considerably less than the 2 orders of magnitude increase observed with STEREO A HET, therefore this trend is not clear. These electron profiles also have similar onset times, with electrons arriving at STEREO A slightly earlier than at Rosetta as expected due to shorter travel distances from the shock.

## 5 Summary

We presented observations of an SEP event detected at Rosetta's target comet 67P, STEREO A and Mars during 6–10 March 2015. At the same time the comet also encountered the Eastern flank of a massive ICME (referred to as *AB* in this study). The Rosetta spacecraft was deep inside the comet coma during the event, which allowed us to study the coma's response. This included an increase in observed electron and ion fluxes which is likely linked to increased ionization rates and possibly increased surface activity; this type of response may affect the production and loss balance of neutral populations. An increase in ionization rates can remove neutral populations, although this is unlikely to be significant at the location of Rosetta in this study; this effect is expected to be more significant at larger distances from the comet. An increase in surface activity (sputtering) on the other hand increases local neutral populations. Increases in He^+^ to alpha particle flux ratios during the event as a result of more charge exchange reactions between the comet's neutral population and solar wind ions indicate an increased neutral population, further pointing toward increased surface activity due to SEP induced sputtering.

The most intense parts of the SEP event at 67P coincided with observations related to the formation of an early stage cometary bow shock (Gunell et al., 2018), suggesting that the SEP event may have enhanced the associated processes. The presence of ICME *AB* together with the SEP event may also have affected the magnetospheric structure, with the cometopause having been pushed further inward toward the nucleus and closer to the observation point (Rosetta's location). Our observations of relatively high energy cometary water group pickup ions are comparable to non‐gyrotropic early phase pickup distributions seen by Giotto at comet 26P/Grigg‐Skjellerup, which was a more active comet when encountered than 67P was during the period studied here. While observations of some pickup ion acceleration were not uncommon throughout the Rosetta mission (Nilsson et al., 2017), water group ions during this SEP event exceeded 1 keV. Such high energy pickup ions are also consistent with changes in the structure of the cometary magnetosphere predicted by modeling.

Furthermore, the electron density recorded by the RPC‐MIP instrument was increased up to 600 cm^−3^ at the onset of the SEP event. Enhanced Langmuir waves, which are usually created via bump‐on‐tail instabilities when a keV electron beam streams through the plasma, were detected coincident with the SEP onset. The presence of SEPs created remotely at ICME shocks may therefore be linked to Langmuir wave activity in cometary environments. It is also possible the SEPs caused extreme surface charging as has been observed on the Moon (Halekas et al., 2009), which can cause surface‐originating electron beams consistent with intense Langmuir wave activity.

We also studied possible sources of the SEP event and discussed its wider relevance in the inner solar system. We demonstrated that considering the gradual and anisotropic nature of the event, its long duration and broad angular spread in the heliosphere, the main particle acceleration source was a massive ICME (labeled *DE* in this study). This ICME emerged from the Sun in the morning of 6 March 2015 and originally consisted of two separate CMEs which later merged. These CMEs are linked to two solar flares with onset times at 04:14 and 06:55 UT on 6 March 2015. The flares may have contributed to the initial production of SEPs, but could not have been the main driver for the entire event. ICME *DE* itself never encountered Rosetta and STEREO A directly; it was observed in situ in the Martian ionosphere on 8 March 2015 (Jakosky, Grebowsky, et al., 2015). Particles were continuously produced at ICME *DE*'s shock for several days. We used ENLIL simulations to show that magnetic field line connections between this production site and STEREO, Mars, and Rosetta were probably present and therefore the SEPs likely propagated along these magnetic field lines to the in situ observation sites. Transport to Rosetta may also have been enhanced by ICME *AB*'s presence in the inner solar system and proximity to 67P.

## Data Availability

ENLIL simulation runs: http://helioweather.net/archive/2015/03/. Mars Odyssey HEND data: NASA's PDS archive https://pds-geosciences.wustl.edu/missions/odyssey/index.htm. Mars Express ASPERA‐3 IMA data: http://rhea.umea.irf.se/∼peje/mex/hk/bkg/. MAVEN data: Planetary Plasma Interactions Node of NASA's PDS database https://pds-ppi.igpp.ucla.edu. Rosetta SREM and RPC data: ESA's PSA https://archives.esac.esa.int/psa/%23%21Table%20View/Rosetta%3Dmission. SOHO LASCO and SDO/AIA images, WIND WAVES radio spectrogram: CDAW Data Center https://cdaw.gsfc.nasa.gov/movie/make_javamovie.php?img1=lasc2rdf&img2=wwaves&date=20150306. SOHO LASCO CME catalog: CDAW Data Center https://cdaw.gsfc.nasa.gov/CME_list/index.html. STEREO data: NASA's Coordinated Data Analysis Web (CDAWeb) database https://cdaweb.gsfc.nasa.gov/index.html/. STEREO SEPT level 2 data: University of Kiel http://www2.physik.uni-kiel.de/stereo/index.php?doc=data.
